# Beyond a Passive Tether: Structural Insights into the Disordered Tail of Hsp90

**DOI:** 10.1021/jacs.6c04189

**Published:** 2026-04-09

**Authors:** Elena Edinach, Angeliki Giannouli, Arina Dalaloyan, Maria Oranges, Debasis Banik, Elian Hadas Yardeni, Annika Elimelech, Michael Sattler, Emmanouil Ntermanakis, Xun-Cheng Su, Daniella Goldfarb

**Affiliations:** † Department of Chemical and Biological Physics, 34976 Weizmann Institute of Science, Rehovot 7610001, Israel; ‡ Department of Chemistry, University of Crete, Heraklion 70013, Greece; § Protein Analysis Unit, Department of Life Sciences Core Facilities, Weizmann Institute of Science, Rehovot 7610001, Israel; ∥ Institute of Structural Biology, Molecular Targets and Therapeutics Center, 9184 Helmholtz Munich, Neuherberg 85764, Germany; ⊥ Bavarian NMR Center and Department of Bioscience, TUM School of Natural Sciences, 12538 Technical University of Munich, Garching 85748, Germany; # State Key Laboratory of Elemento-Organic Chemistry, College of Chemistry, Nankai University, Tianjin 300071, China

## Abstract

The molecular chaperone Hsp90 is an abundant and essential homodimer that supports the stability and folding of hundreds of client proteins in cells. Its C-terminal domain (CTD) mediates dimerization and serves as a docking site for cochaperones bearing tetratricopeptide repeat (TPR) domains, which recognize the conserved MEEVD motif located at the end of an intrinsically disordered CTD tail. Despite its conservation, the structural role of this tail remains poorly understood. We investigated the conformational behavior of the yeast Hsp90 (yHsp90) CTD tail and its response to the binding of the TPR-containing PPIase cochaperone Cpr6. Using site-directed spin labeling with nitroxide and Gd­(III) labels, we examined full-length yHsp90 (FL), isolated CTD (IsoC), and tail-truncated variants by double electron–electron resonance (DEER) and EPR spectroscopy. The CTD tails in IsoC and FL adopted distinct conformational ensembles, attributed to intramolecular interactions with the middle domain in FL. Cpr6 binding abolished these differences, indicating disruption of intramolecular tail contacts. In IsoC, the tail also stabilized an additional CTD conformation near the dimer interface that was absent in FL and was lost upon tail truncation, consistent with tail–CTD interactions observed by NMR. This population was reduced upon Cpr6 binding, shifting the IsoC conformational distribution toward that of FL. Additionally, this population was reduced in cellular environments and mimics. Together, our results demonstrate that the disordered CTD tail is an active structural element that modulates both its own conformational ensemble and CTD architecture, highlighting its potential functional relevance in the Hsp90 chaperone cycle.

## Graphical abstract

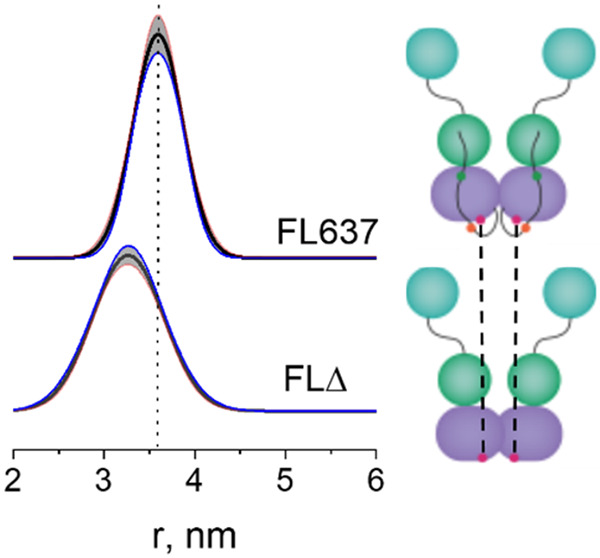

## Introduction

The molecular chaperone heat shock protein 90 kDa (Hsp90) is an abundant and essential protein required for the stability and/or folding of hundreds of client proteins, many of which are implicated in various types of cancer.^2^ Structurally, Hsp90 is a flexible homodimer with each protomer comprising three highly conserved domains: the amino-terminal domain (NTD), which contains the ATP hydrolysis site (ATPase),^3^ the middle domain (MD), which is important for ATP hydrolysis and client binding; and the carboxyl-terminal domain (CTD), which mediates dimerization of the two monomers, and serves as a binding site for cochaperones (Figure 1A).^4–6^ Hsp90 functions through an ATP-hydrolysis driven conformational cycle that involves transitions between open and closed states and association with cochaperones. Although recent cryo-electron microscopy (cryo-EM) studies have captured several nucleotide- and cochaperone–bound complexes and provided valuable mechanistic insights,^7–12^ some key aspects of the cycle remain unresolved, as recently reviewed.^13^

**Figure 1:**
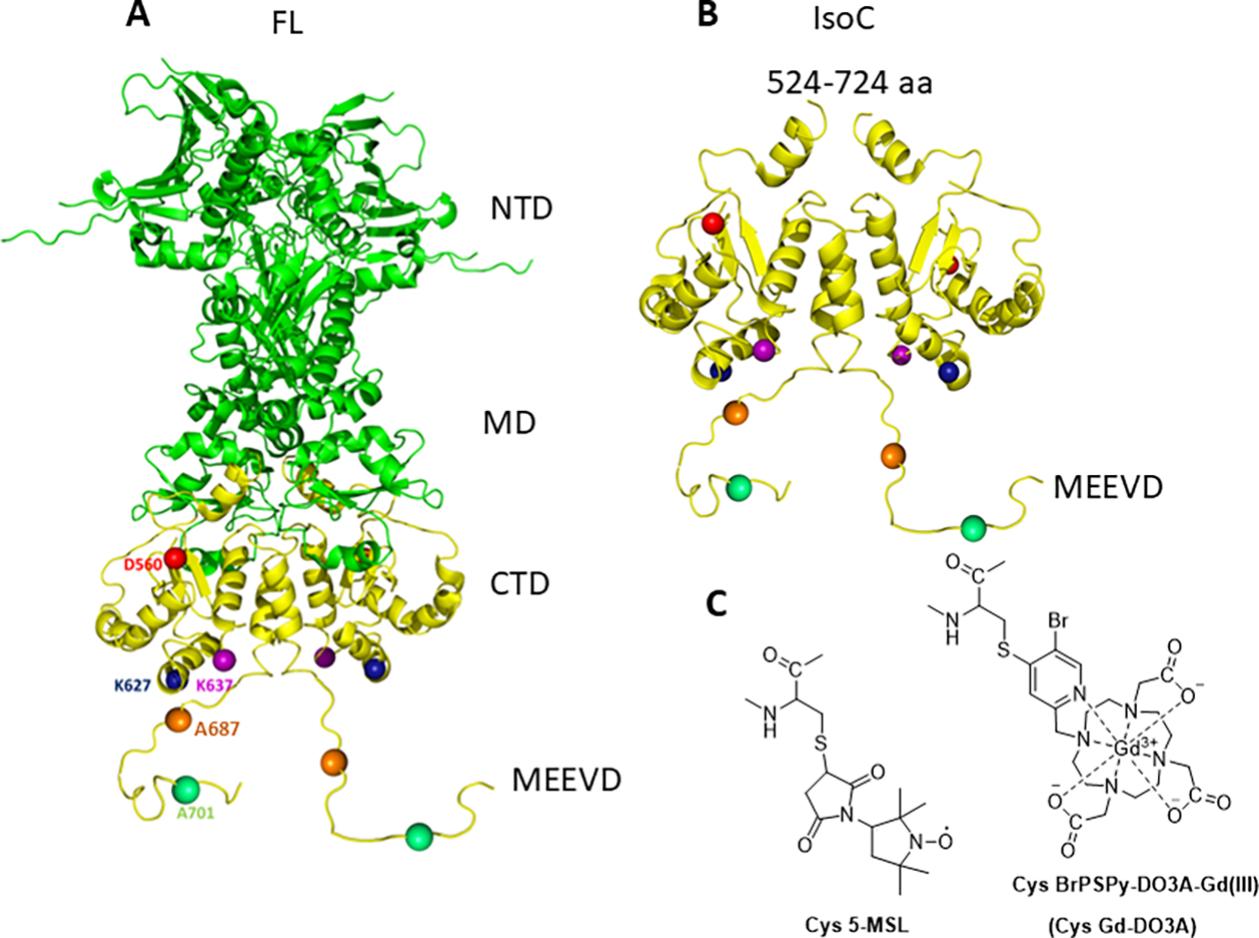
(A) X-ray structure of full-length yHsp90 in the presence of the NTD cochaperone Sba1 (omitted here for clarity, PDB 2CG9 ^27^). In yellow are shown the CTDs, and the spin-labeled positions are indicated with spheres. The disordered CTD tail is drawn arbitrarily. (B) Same as in (A), showing only the IsoC part. (C) Structures of the spin labels used in this work after conjugation to a cysteine residue.

In the absence of nucleotide, that is apo-state, Hsp90 adopts the so-called “open” conformation, in which the NTDs are not dimerized and their relative positions are disordered, resulting in a broad distribution of inter-NTD distances. Upon ATP binding, the NTDs undergo internal rearrangements that promote their association, leading to the formation of a closed conformation.^14–16^ Furthermore, ATP hydrolysis to ADP in the absence of substrate and/or cochaperones gives rise to an even more compact state.^17^ In the presence of the NTD cochaperone Sba1, the NTDs adopt an even more tightly associated “packed” conformation.^18^ Notably, in these NTD conformations, the CTDs remain dimerized^14,17,18^ with the strength of the dimerization only moderately modulated by the presence of the MDs and NTDs.^19^

One of the least resolved structural elements of Hsp90 is the intrinsically disordered CTD tail. Intrinsically disordered terminal tails are increasingly recognized as important modulatory elements in protein function rather than passive appendages. Such regions may engage in transient intramolecular interactions, act as entropic regulators of domain accessibility, and respond sensitively to binding partners or post-translational modifications. Well-established examples include histone N-terminal tails, which regulate chromatin organization,^20^ and disordered regulatory segments in kinases and ion channels that bias conformational equilibria or control partner binding.^21–23^ In addition, and relevant to Hsp90 is the unstructured tail of the Hsp90 cochaperone p23 which regulates the Hsp90 ATPase activity.^24^ In Hsp90, the CTD tail contains the conserved MEEVD motif, which mediates interactions with tetratricopeptide repeat (TPR) containing cochaperones such as Sti1/Hop and Cpr6 ^14,16^ which modulate Hsp90 conformation and/or ATPase activity.^25^ While the MEEVD motif itself can sometimes be visualized as a short helix when bound to TPR proteins,^12,26^ the remaining 30+ residues of the CTD tail are consistently absent from cryo-EM and crystallographic reconstructions due to their high disorder and conformational heterogeneity. As a result, the structural and regulatory roles of this region remain largely unexplored.^27^

Despite its established role in recruiting TPR-containing cochaperones, it remains unclear whether the CTD tail functions merely as a flexible tether for cochaperone recruitment, analogous to a fishing rod with a disordered line and a MEEVD “hook”,^28^ or whether it also engages in intramolecular interactions with other Hsp90 domains, thereby influencing CTD structure at the dimer interface and eventually contributing to regulation. To address these questions, we investigated the conformation of the disordered CTD tail of yeast Hsp90 (yHsp90) and its influence on the folded CTD, both in the apo state and upon binding of the CTD cochaperone Cpr6. Cpr6 is a cyclophilin-family peptidyl-prolyl isomerase (PPIase) in *Saccharomyces cerevisiae* that participates Hsp90s functional cycle.^16,29^ During client transfer from Hsp70 to Hsp90, the TPR-containing cochaperone Sti1 (Hop) binds the CTD and inhibits N-terminal dimerization and ATPase activity^30^ before being displaced by Cpr6, which restores ATPase activity and stabilizes closed conformations.^25,31–33^

To probe the solution-state behavior of the CTD tail, we prepared full-length (FL) yHsp90, isolated CTD (IsoC), and their tail-truncated variants (FLΔ and IsoCΔ).^33^ Using site-directed spin labeling with nitroxide and Gd­(III) labels at positions within the folded CTD and the disordered tail, we employed double electron–electron resonance (DEER) spectroscopy to quantify intermonomer distances and continuous-wave (CW) EPR to assess residue-specific dynamics. We additionally used NMR spectroscopy to detect interactions between the CTD tail and the folded CTD. Finally, we examined the conformational behavior of FL and IsoC constructs in the presence of Cpr6 and in physiologically relevant environments, i.e., in yeast and HeLa cell extracts, and inside HeLa cells.

## Results and Discussion

### Domain-Dependent CTD Tail Conformations in apo and Cpr6-Bound yHsp90

Three different positions in the folded part of the CTD (variants D560C, K627C, and K637C) and two positions in the disordered CTD tail (variants A687C and A701C) (Figure 1A,B) in IsoC and FL were labeled with a Gd­(III) (Gd-DO3A) and a nitroxide spin label (MSL) (Figure 1C). The sequences of the variants used are given in Section S1 in the SI, and a list of all Hsp90 variants is given in Table S1. The purity of the proteins was confirmed by Sodium Dodecyl Sulfate Polyacrylamide Gel Electrophoresis (SDS-PAGE) and mass spectrometry (Figures S1 and S2).

Before addressing the conformational ensemble of the CTD tail in the presence of Cpr6, we ensured binding of Cpr6 to IsoC and FL by measuring the Cpr6-yHsp90 dissociation constant, *K*_d_, with surface plasmon resonance (SPR) using both direct and competitive approaches. This was done in the presence and absence of the spin label. All SPR measurements were done in the absence of nucleotides, revealing binding with *K*_d_ values in the range of 0.25–1.5 μM; data are shown in Figure S3, and the derived *K*_d_ values are listed in Table S2. For FL637, measurements were also carried out in the presence of Mg^2+^ and AMPPNP, and no effect on the *K*_d_ was detected. The *K*_d_ values obtained for FL are comparable to those reported for FL yHsp90 (0.24 μM)^25^ determined with ITC, but larger than those reported by SPR competition experiments (0.014–0.026 μM).^34^

#### DEER Distance Measurements

After establishing the binding of Cpr6 to IsoC and FL yHsp90, we investigated the conformation of the CTD tail in the presence and absence of Cpr6. We performed DEER distance measurements between spin labels introduced at two different positions along the flexible tail, A687C located near the beginning of the CTD tail, and A701C, situated only four residues downstream of the conserved MEEVD motif. The DEER measurements report interprotomer distances within the Hsp90 dimer, i.e., A687C(1)–A687C(2) or A701C(1)–A701C(2). We used the Gd-DO3A spin label (Figure 1C) whose rigid tether minimizes linker flexibility, such that the width of the resulting distance distributions primarily reflects conformational disorder of the protein rather than label mobility.^35^ In addition, Gd-DO3A is stable under cellular conditions.^36^

The DEER distance distributions obtained for IsoC-A687C and FL-A687C labeled with Gd-DO3A (hereafter IsoC687-Gd and FL687-Gd) are shown in Figure 2. In both cases, the distance distributions exhibit widths comparable to those typically observed for folded regions. Given the proximity of residue 687 to the structured CTD, this suggests that the proximal portion of the tail adopts a partially ordered conformation. Notably, the most probable distance (corresponding to the maximum of the distribution) is longer for IsoC687-Gd (5.71 nm) than for FL687-Gd (5.05 nm) and upon Cpr6 binding, the IsoC687-Gd distance shortens to 5.13 nm (Figure 2A). In contrast, Cpr6 binding to FL687-Gd, either in the presence or absence of Mg^2+^/AMPPNP, does not measurably affect the distance distribution (Figure 2B), which remains with a maximum at ∼5.1 nm. Small variations of 0.05–0.15 nm are within experimental uncertainty. Interestingly, Cpr6 binding abolishes the difference between IsoC and FL at position 687: in the presence of Cpr6, IsoC687-Gd and FL687-Gd exhibit essentially identical distances.

**Figure 2:**
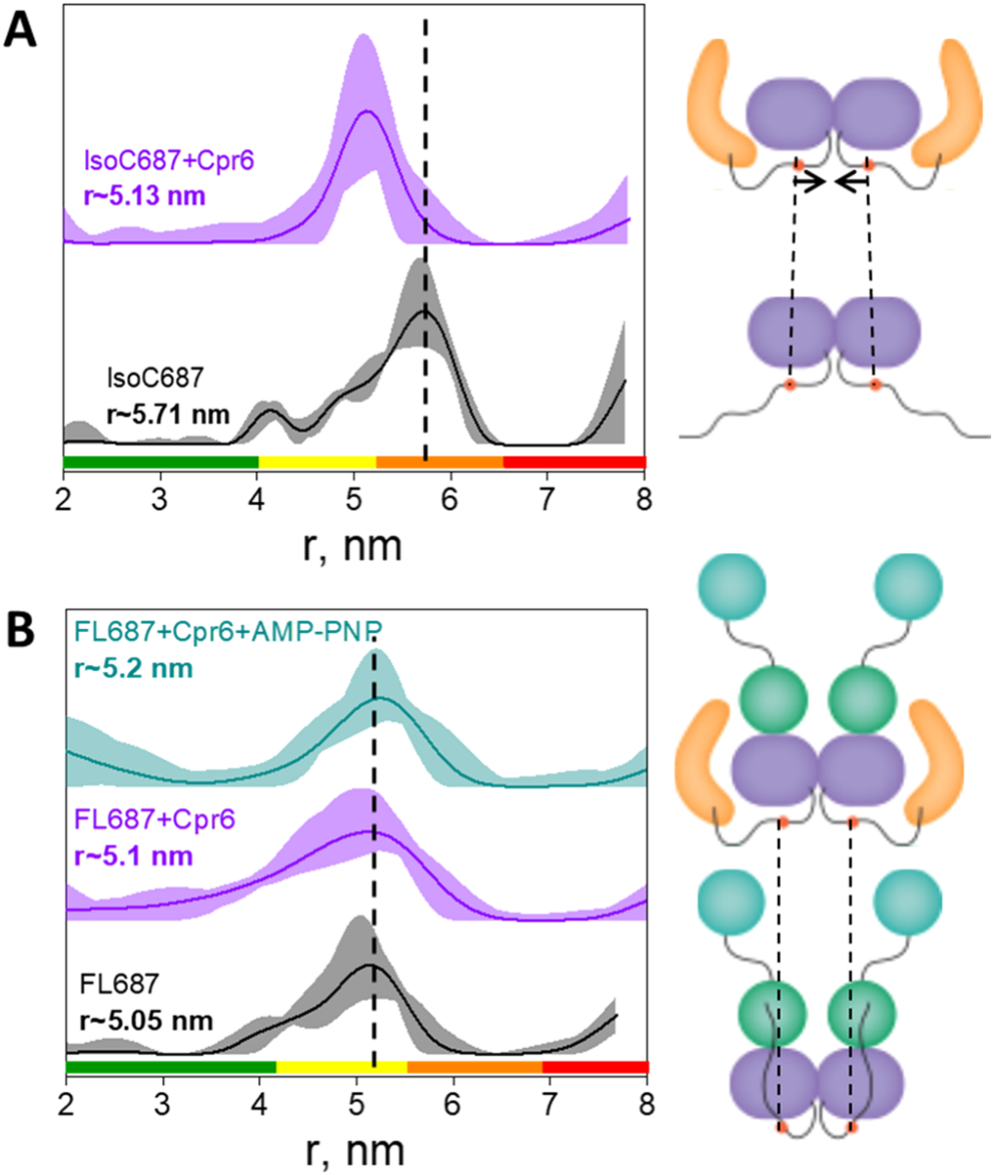
DEER distance distributions with confidence limits for (A) IsoC687-Gd with and without Cpr6 and (B) FL687-Gd with and without Cpr6 and with Cpr6 in the presence of Mg^2+^/AMPPNP. Data analysis was done using Tikhonov regularization.^37^ Color bars on the *X*-axis denote reliability of the distance distribution as follows: green= shape reliable; yellow= mean and width reliable; orange= mean reliable; red= nonreliable.^37^ The primary DEER data with the background correction function and the background-corrected data with their fit are shown in Figure S5. On the right side of each panel, we present schematic illustrations of IsoC687 and of FL687 with all three domains in the open conformation of the NTDs (light blue). The orange dots represent the Gd­(III) label on the tail, and the vertical lines highlight the changes in their distance upon binding of Cpr6, shown as a yellow banana. The tail representations are intended solely as schematic illustrations, showing two limiting scenarios: a predominantly free-hanging ensemble and an ensemble that transiently engages folded regions of Hsp90.

DEER measurements performed on IsoC701-Gd and FL701-Gd did not yield any detectable modulations (Figure S4). Given the high labeling efficiency confirmed by mass spectrometry (Figure S2), this indicates that the interprotomer distance at position 701 exceeds the DEER detection range. For IsoC701-Gd, binding of Cpr6 does not induce any detectable change in the DEER trace, whereas for FL701-Gd, Cpr6 binding introduces a noticeable bend in the DEER time trace, consistent with a shortening of the interspin distance. A quantitative analysis was not pursued due to the difficulty of reliably separating intra-molecular signal modulation from background decay.

Together, the DEER measurements with the label at positions 687 and 701 demonstrate that the conformational ensemble of the CTD tail differs between apo-IsoC and apo-FL yHsp90. This could be due to different interactions of the tail in IsoC and FL with the folded Hsp90 domains, possibly the folded part of the CTD and the MD (in the case of FL). Furthermore, Cpr6 binding induces distinct conformational changes in the tail: it shortens the interprotomer distance at position 687 in IsoC to match that of FL, and it reduces the distance at position 701 in FL. Collectively, these findings indicate that Cpr6 binding to the MEEVD motif promotes a more compact conformational ensemble of the CTD tail. These results are illustrated schematically in Figure 2.

To place our DEER-derived structural information in a broader structural context, we performed AlphaFold predictions for apo-IsoC and apo-FL yHsp90 and in complex with Cpr6. Although AlphaFold can identify disordered regions based on a low Predicted Local Distance Difference Test (pLDDT) value (<50), which is a per-residue confidence score that estimates how reliable the predicted local structure is, it does not explicitly model the heterogeneous conformational ensembles characteristic of intrinsically disordered segments.^38,39^ Nevertheless, because the distance distributions for a label at position 687, which is close to the folded CTD, are relatively narrow, these data may provide a meaningful benchmark for evaluating AlphaFold predictions.

Isothermal titration calorimetry reported a 1:1 stoichiometry corresponding to two Cpr6 molecules per Hsp90 dimer^25^ and therefore we used 1:1 complex in the AlphaFold predictions. As no experimental structures of Cpr6 or its complex with yHsp90 are currently available, these predictions are the only structural reference. For IsoC in complex with Cpr6, different AlphaFold runs generated models with variable Cpr6 orientations, therefore these predictions were not considered further. For each AlphaFold run, the five resulting models were retained, and their superimposed structures are shown in Figure S6A. For apo FL, the model predicts a closed Hsp90 conformation, which is not the dominant state in the absence of nucleotides. However, because CTD dimerization is largely independent of nucleotide binding,^17^ we used the predicted full-length structures as a reasonable structural reference of the CTD for subsequent analyses. To compare with experimental results, Gd­(III) spin labels were added in silico using MtsslWizard,^40^ and distance distributions were calculated for each model. The distributions were then summed into a single distribution (Figure S6B,C). These distributions are in the range of the experimental data and reproduce the slightly longer distance observed experimentally for apo-IsoC vs FL (5.7 nm vs 5.05 nm). For the label at position 701, AlphaFold predicted distances of ∼12 nm for FL and ∼10 nm for IsoC, which fall outside the DEER detection range, and a shortening of the FL701 distance upon binding of Cpr6, in qualitative agreement with the experimental observations.

#### Residue-Specific Dynamics by CW-EPR

Beyond interdomain distance measurements, we probed the local dynamics of the CTD tail using nitroxide spin labeling combined with CW-EPR. This approach is sensitive to residue-specific motional freedom and enables us to detect differences in the tail’s conformational ensembles and their response to Cpr6 binding. Residues A687C and A701C in IsoC and FL were labeled with MSL (Figure 1C), and room-temperature X-band CW-EPR spectra were recorded in the absence and presence of Cpr6 (Figure 3). The CW-EPR spectrum of IsoC687-MSL is characteristic of a spin label undergoing fast motion on the EPR time scale. Upon addition of a 4-fold molar excess of Cpr6, additional spectral features appeared at 334.3 and 340.9 mT, indicative of a population with a restricted motional freedom. Such a restriction reflects increased local rigidity and/or transient contacts of the labeled residue. Spectral simulations (Figure S7) show that this slow-motion component accounts for 31.3% ± 6.5% of the total protein population. A qualitatively similar response was observed for FL687-MSL. In contrast to IsoC, however, a slow-motion component was already present in the absence of Cpr6, comprising 13.5% ± 7.0% of the protein molecules. Upon Cpr6 binding, this fraction increased to 34.1% ± 3.6%, comparable to that observed for IsoC687-MSL in the presence of Cpr6. For IsoC701-MSL, the CW-EPR spectra exhibited only a single, fast-motion component, both with and without Cpr6. In contrast, FL701-MSL displayed a substantial slow-motion population already in the absence of Cpr6 (35.9%), which further increased to 54.5% upon Cpr6 binding. The Cpr6-induced increase in the slow-motion component for all variants is summarized in Figure S7D.

**Figure 3:**
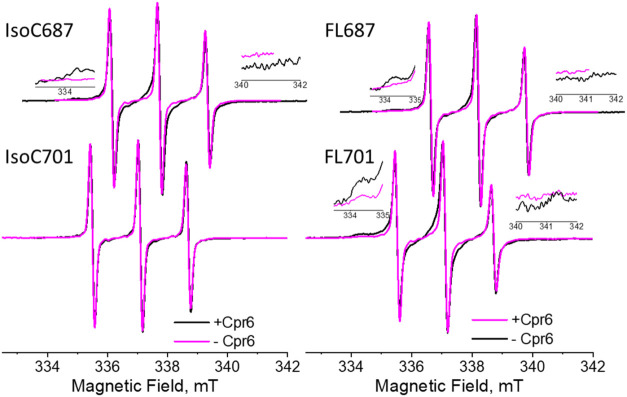
Room temperature, normalized X-band CW-EPR spectra in the presence and absence of Cpr6 for the 687 and 701 variants (indicated). The inserts zoom in on the region where the signatures of the slow motion spectrum are. The magnetic field scale is the same for all spectra; the top spectra were shifted by 1 mT for clarity. The parameters of simulations are given in Table S3.

To further assess Cpr6 binding stoichiometry, titration experiments with increasing amounts of Cpr6 were performed for FL687-MSL and IsoC687-MSL (Figure S8). For FL687-MSL, the contribution of the slow-motion component saturated at a 1:1 molar ratio of yHsp90 monomer to Cpr6, consistent with binding of two Cpr6 molecules per yHsp90 dimer. For IsoC687-MSL, the smaller amplitude of the restricted-motion component and the resulting lower signal-to-noise ratio precluded unambiguous conclusions regarding stoichiometry.

Taken together, the CW-EPR results indicate that the MSL spin label at positions 687 and 701 in IsoC experiences very high mobility, consistent with a largely unconstrained tail. In contrast, the corresponding positions in FL populate conformations with restricted spin-label motion, suggesting transient interactions with other regions of Hsp90, most likely the folded CTD and/or the MD, which are in spatial proximity to the tail. Notably, the label positioned closer to the distal end of the tail (position 701) exhibited a larger fraction of restricted-motion conformations, suggesting preferential interactions of the end region of the tail with the MD, while regions closer to the CTD preferentially contact the CTD itself. This is consistent with the DEER data discussed above (Figure 2). The hypothesis that the tail in FL Hsp90 interacts with residues in the MD is supported by the fact that the disordered tail is highly negative with a total charge of −20, while strong positive patches in the M-domain can be found in residues 317–351 (10 lysines and arginines), 384–403 (6 residues), and 435–450 (4 residues).

Binding of Cpr6 increased the population of the restricted-motion conformations at both labeling sites in FL, consistent with the formation of new tail–Cpr6 interactions. In IsoC, where interactions between Cpr6 and the MD are absent, Cpr6 binding reduced tail mobility near the CTD but had little effect toward the distal end of the tail. This observation suggests that Cpr6 is not stably docked to the folded CTD in IsoC, which may underlie the structural heterogeneity observed in AlphaFold predictions of the IsoC–Cpr6 complex.

The CW-EPR measurements show that the intrinsically disordered CTD tail adopts distinct conformational ensembles in IsoC and FL, consistent with the DEER results. In FL, a subset of tail conformations exhibits restricted local dynamics, consistent with interactions with the folded CTD and/or the MD, whereas the tail in IsoC remains highly dynamic. Binding of Cpr6 restricts tail mobility near the CTD in IsoC but does not affect the distal tail region, underscoring fundamental differences in how the CTD tail engages with Cpr6 in IsoC and FL. We hypothesize that these differences are driven by interactions between the tail and the MD in the full-length protein.

### The CTD Tail Modulates the Structure of the Folded CTD Region

To determine whether the disordered tail and Cpr6 binding influence the structure of the folded CTD dimer, we performed DEER measurements on the Gd-DO3A–labeled variants D560C, K627C, and K637C, where the label is within the structured CTD. No differences were observed between IsoC and FL at positions D560C and K627C, and Cpr6 binding had no effect on the corresponding intermonomer distance distributions (Figures S9 and S10), indicating that these regions of the CTD are structurally insensitive to both tail context and Cpr6 binding. In contrast, position K637C, located near the CTD dimer interface and adjacent to the disordered tail, displayed marked differences. IsoC637-Gd exhibited two distance distributions, one with a maximum at 3.4 nm and a broader one centered at 4.9 nm (Figure 4A), whereas FL637-Gd showed a single peak at 3.6 nm that remained unchanged upon Cpr6 binding (Figure 4B). The shorter distances (3.4–3.6 nm) are in excellent agreement with those predicted from the crystal structure of full-length yHsp90 in complex with Sba1 (PDB 2CG9).^17^ Importantly, increasing concentrations of Cpr6 selectively reduced the population of the longer-distance component in IsoC637-Gd (Figure 4C,D), indicating that Cpr6 binding stabilizes the more compact CTD conformation and suppresses structural heterogeneity.

**Figure 4:**
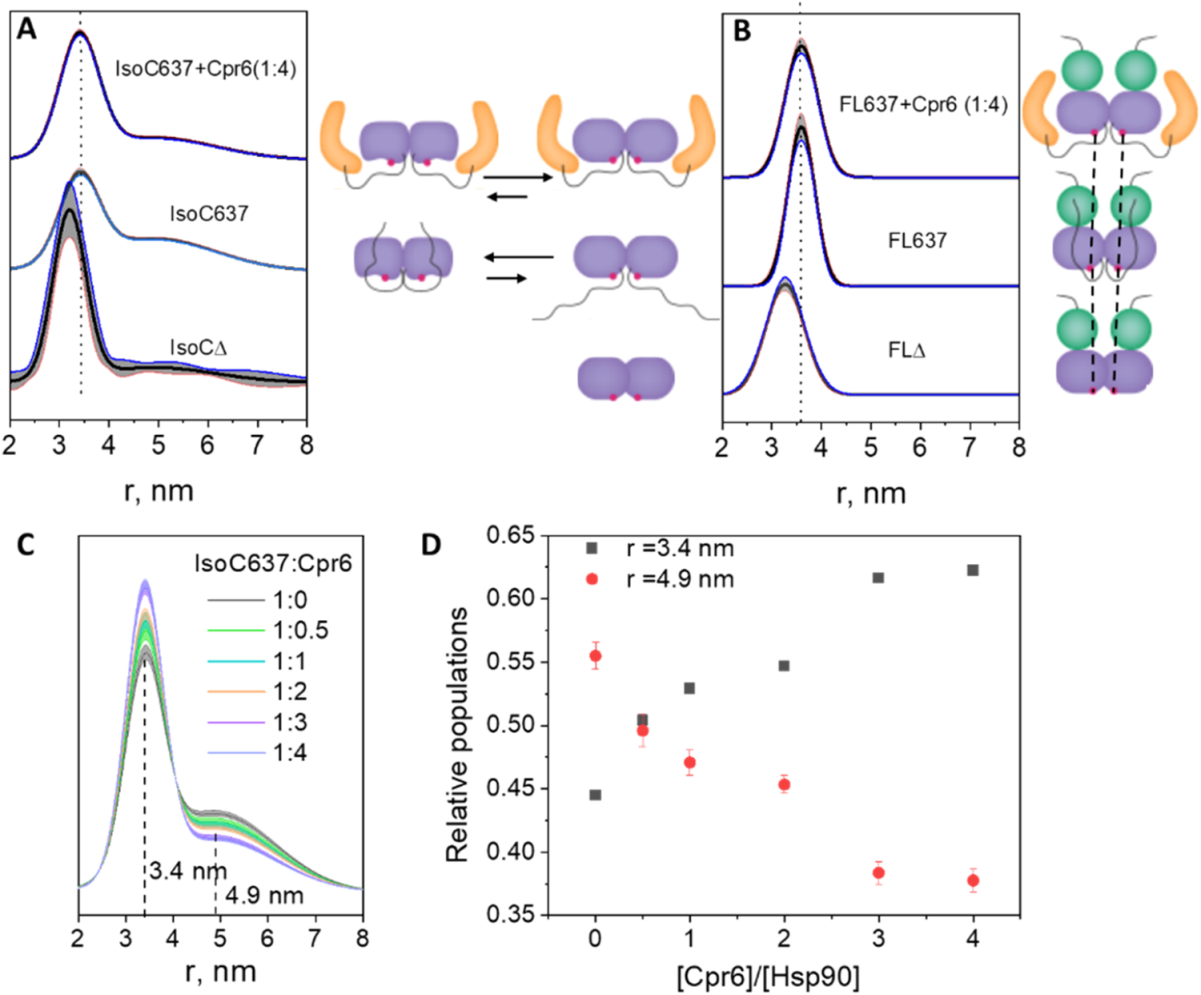
DEER distance distributions with confidence limits for (A) IsoC637-Gd+Cpr6(1:4), IsoC637-Gd, and IsoCΔ-Gd and (B) FL637-Gd+Cpr6(1:4), FL637-Gd, and FLΔ-Gd. The dotted vertical line is added to highlight detected shifts, (C) IsoC637-Gd with increasing molar ratio of Cpr6 (indicated). The protein concentration in all samples was 100 μM. The DEER data of IsoC637-Gd and FL637-Gd were analyzed globally using GLADDvu^40^ and the primary DEER data and their fit are shown in Figure S11. The data for IsoCΔ-Gd and FLΔ-Gd were analyzed individually. (D) The relative amounts of the two distance populations for IsoC637-Gd as a function of the relative amount of Cpr6. On the right side of panels (A) and (B) we present schematic illustrations of IsoC637 and FL637 (without the NTD due to space constraints) with the Gd­(III) label at position 637 shown in red. For IsoC, changes in equilibrium between the two populations with and without Cpr6, and the disappearance of the population with the long distance upon the removal of the tail are shown. For FL637 one population is present both in the presence and absence of Cpr6, whereas the tail removal reduced the distance between the Gd­(III) labels.

We hypothesize that the differences between IsoC and FL originate from distinct intramolecular interaction patterns of the disordered CTD tail. In IsoC, the tail remains unconstrained due to the absence of MD and can transiently interact with the folded CTD, thereby promoting a more heterogeneous conformational ensemble. In contrast, in the full-length protein, tail interactions with the MD limit its engagement with the CTD, resulting in a more uniform CTD conformation.

To directly test the contribution of the tail to the observed CTD heterogeneity, we generated tail-truncated variants of IsoC637 and FL637 (IsoCΔ and FLΔ, respectively), labeled them with Gd-DO3A, and performed DEER measurements (Figure 4A,B). In IsoCΔ-Gd, the longer-distance population at 4.9 nm was strongly reduced, and the remaining peak shifted from 3.4 to 3.2 nm, indicating compaction of the CTD dimer. Similarly, tail truncation in FLΔ-Gd shortened the intermonomer distance from 3.6 to 3.3 nm, yielding a distance nearly identical to that observed for IsoCΔ-Gd. These results demonstrate that the disordered tail modulates the CTD conformation in both IsoC and FL and that its removal stabilizes a compact CTD architecture. Notably, Cpr6 binding and tail removal drive IsoC toward the same structural end point, characterized by a rigid and compact CTD conformation at position 637, proximal to the tail attachment site. This behavior is summarized schematically in Figure 4A,B.

To identify direct tail–CTD contacts that could account for the DEER-observed heterogeneity in IsoC, we performed NMR (^1^H–^15^N HSQC) measurements comparing IsoC and IsoCΔ. The resulting chemical shift perturbations revealed specific regions affected by the presence of the tail, most prominently residues 560–575 and around residue 620 (Figure 5). These perturbations indicate transient intramolecular interactions between the disordered tail and defined surfaces of the folded CTD, providing a molecular basis for the additional conformational population observed in IsoC.

**Figure 5:**
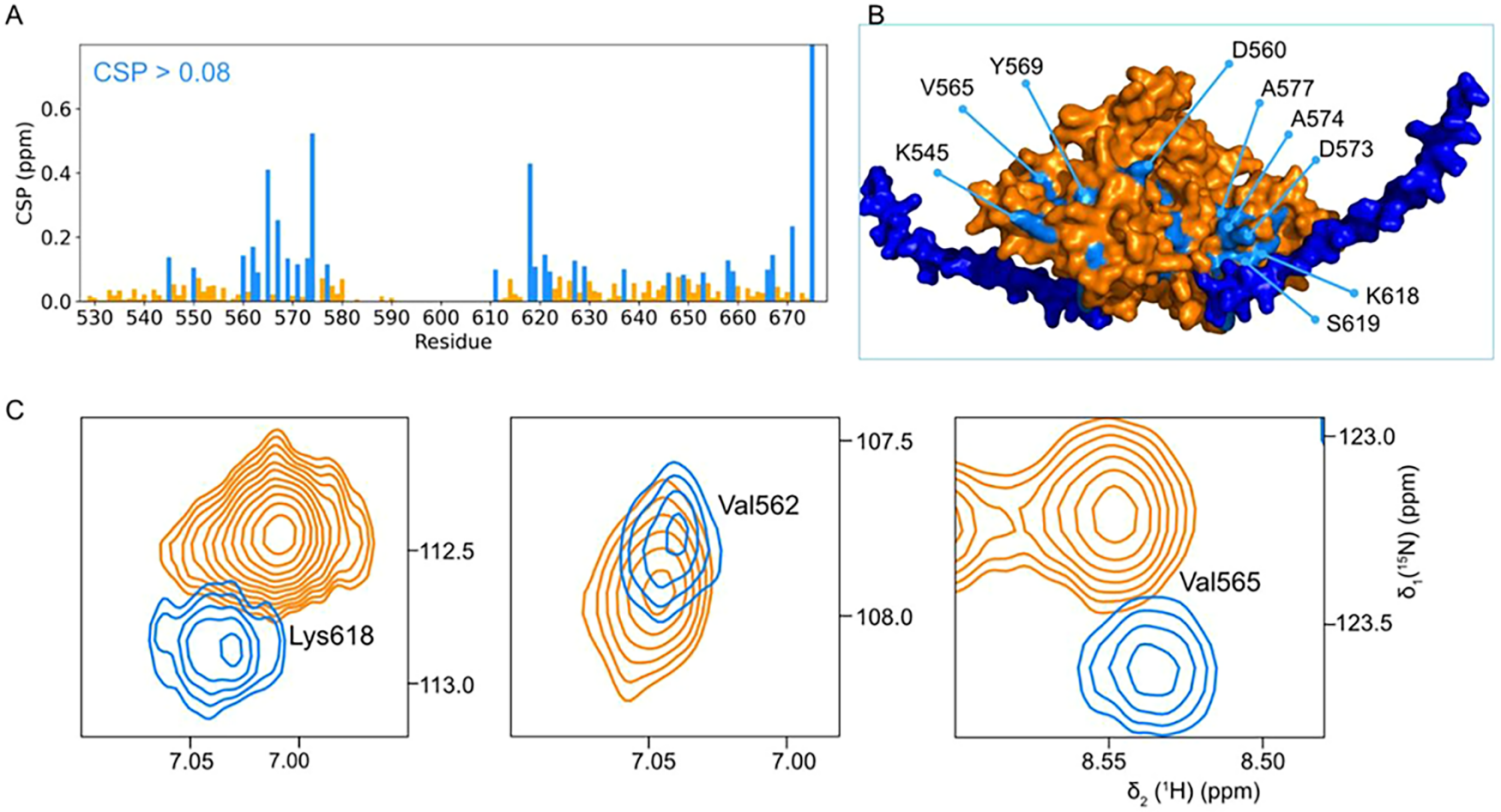
(A) CSP plot of IsoC as a consequence of the tail removal, (B) AlphaFold model of the CTD (orange), including the C-terminal tail (dark blue), depicting the CSPs region from plot A on the structure of the CTD. Residues with CSPs > 0.08 are shown in sky blue. (C) Zoomed-in views of the ^1^H^15^N HSQC spectrum of IsoCΔ (orange) and isoC (skyblue) showing residues perturbed by tail contacts. The full spectrum is shown in Figure S12.

To assess whether the structural differences observed for IsoC637 and FL637, as well as their tail-truncated variants, are accompanied by changes in dimer stability, we performed differential scanning fluorimetry (DSF) measurements (Figure S13). IsoC unfolded at a higher temperature than FL, and removal of the disordered CTD tail resulted in a modest increase in thermal stability, reflected by an increase in the melting temperature (*T*_m_) of ∼2 °C relative to the corresponding full-length constructs. Importantly, these effects were small, indicating that the overall dimer stability is comparable among all 637 variants. We also carried out microscale thermophoresis­(MST) measurements to see whether the CTD disordered tail influences the dimer’s dissociation constant, *K*_d‑dim_. The results, presented in Figure S14, and the *K*_d‑dim_ values given Table S4 reveal that *K*_d‑dim_ for IsoC is consistently higher than for FL, as reported earlier,^19^ stressing the contribution of the MD to the stability of the dimers. Overall, we did not detect a significant effect of the disordered tail on the stability of the dimers.

Finally, as DEER has been shown to allow the extraction of binding constants,^41,42^ we used the DEER-derived population shifts in IsoC637 upon Cpr6 binding (Figure 4D) to estimate the affinity of Cpr6 for the two observed CTD conformations. We considered a model in which Cpr6 binds independently and noncooperatively to each monomer, with distinct affinities for conformations *a* and *b* (see SI and Figure S15). This model provided a good fit to the data, yielding *K*_d,a_ = 158 ± 32 μM and *K*_d,b_ = 528 ± 105 μM. An apparent discrepancy exists between these values and the single dissociation constant obtained from the SPR measurements (∼1 μM). This difference likely arises from the measurement temperatures: SPR measurements were conducted at room temperature, whereas DEER data were collected at 10 K. The DEER-derived populations reflect the conformational ensemble at the time of freezing, estimated at ∼265 K.^43^ As a result, the *K*_d_ values obtained by SPR and DEER cannot be directly compared.^44,45^

Assuming that the temperature range of 265–298 K lies within the van’t Hoff linear regime, we estimated Δ*H* ^o^ and Δ*S* ^o^ of the binding of Cpr6 to IsoC from the temperature dependence of *K*_d_. This gave for *K*_d,a_ = 158 ± 32, Δ*H* ^o^ = 24.1 kcal/mol and Δ*S* ^o^ = 0.1 kcal/mol·K, consistent with an endothermic, entropy-driven reaction. While the estimated Δ*H* ^o^ value appears relatively high, similar thermodynamic behavior has been reported for Ca^2+^-bound calmodulin upon peptide binding^46^ and for apo-calmodulin with nitric oxide synthase II-derived peptides.^47,48^ A lower estimate of the freezing temperature would lower Δ*H* ^o^.

Together, the results presented in this section demonstrate that the disordered CTD tail actively modulates the conformational ensemble of the folded CTD rather than acting as a passive appendage or modulating dimer strength or stability. In IsoC, tail–CTD interactions introduce additional structural heterogeneity at the dimer interface, leading to distinct CTD conformations absent in the full-length protein. In the context of full-length Hsp90, engagement of the tail with the MD is likely to limit its interaction with the CTD, thereby stabilizing a more uniform and compact CTD architecture. Importantly, either tail removal or Cpr6 binding suppresses tail-mediated heterogeneity in IsoC and drives the CTD toward a common, compact structural end point.

#### IsoC Becomes More Compact in Cell Extracts and in Cells

We next examined whether the behavior observed for IsoC637-Gd and FL637-Gd upon Cpr6 binding in vitro also occurs in a cellular context, where Hsp90 interacts with endogenous TPR-containing partners. We first performed DEER measurements on IsoC637-Gd and FL637-Gd in the presence of yeast cell extracts and resulting distance distributions mirrored those obtained in solution upon Cpr6 binding (Figure 6A,C). Specifically, for IsoC637-Gd, the population corresponding to the longer-distance component at ∼4.9 nm was reduced (Figure 6A), whereas no change was observed for FL637-Gd (Figure 6C). These results suggest that endogenous TPR-domain cochaperones present in the extract reproduce the conformational effects observed upon Cpr6 binding to IsoC637-Gd in vitro. We then asked whether this behavior persists in living cells. Because of technical challenges associated with protein delivery and freezing yeast spheroplasts, we performed in-cell DEER measurements in human HeLa cells following electroporation delivery^49^ of the labeled constructs. Human and yeast Hsp90 share 61% sequence homology, supporting the relevance of this heterologous system. Prior to in-cell EPR experiments, efficient intracellular delivery of IsoC637 and FL637 was verified by labeling the proteins with the fluorescent dye ATTO488 and imaging by fluorescence microscopy (Figure S17). Both constructs were successfully delivered, with IsoC637 showing higher uptake efficiency, probably due to its smaller size. Using the same delivery conditions, IsoC637-Gd and FL637-Gd were introduced into HeLa cells for DEER measurements.

**Figure 6:**
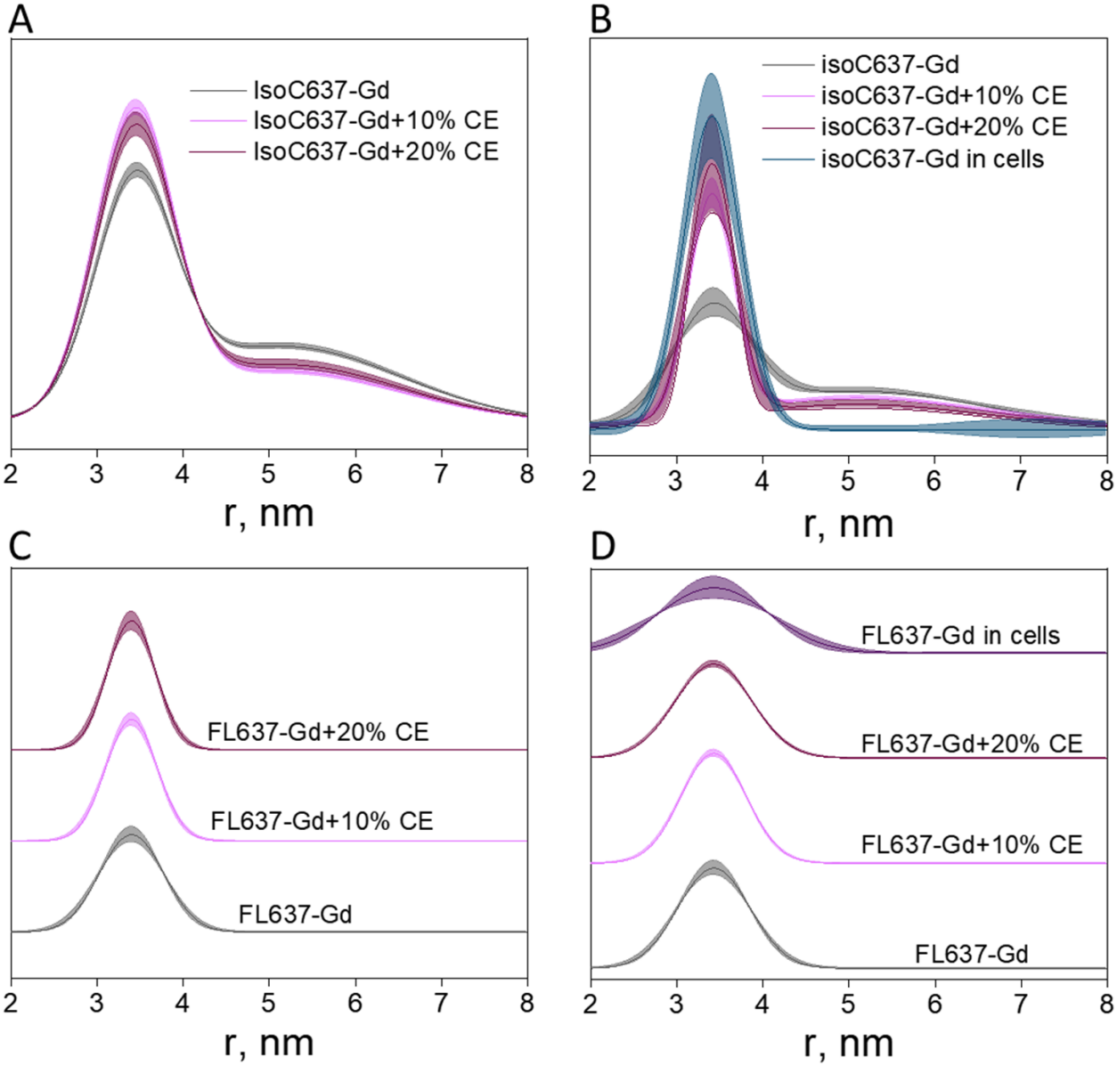
Normalized DEER distance distributions of IsoC637-Gd (top) and FL637-Gd (bottom) with (A, C) yeast cell extracts and (B, D) HeLa cell extracts and HeLa cells. The data were analyzed by global fit using GLADDvu. The corresponding time-domain data and their fits are shown in Figure S16.

In-cell DEER data revealed the complete disappearance of the broad ∼4.9 nm distance population for IsoC637-Gd (Figure 6B), consistent with a pronounced compaction of the CTD dimer in the cellular environment. In contrast, the distance distribution of FL637-Gd remained unchanged (Figure 6D). Similar results were obtained when the labeled proteins were mixed with HeLa cell extracts, where a reduction of the long-distance component was again observed for IsoC637-Gd but not for FL637-Gd (Figure 6B,D). Thus, both cell extract and in-cell measurements recapitulated the conformational behavior observed in vitro in the presence of Cpr6.

Overall, these findings demonstrate that the compaction of IsoC observed upon Cpr6 binding in vitro also occurs in complex cellular environments, strongly suggesting that endogenous TPR-domain proteins reproduce Cpr6 effects. Notably, this modulation is observed not only in yeast extracts but also in human cells, underscoring the conserved nature of CTD tail–mediated interactions across species.

To assess whether IsoC687-Gd retains its ability to engage TPR-domain proteins in a complex cellular environment, and to test whether the conformational changes observed by DEER can arise from such interactions, we performed two independent pull-down experiments followed by mass spectrometry using IsoC687-Gd as bait in HeLa cell extracts. We chose Hela cell extracts because in-cell measurements were carried out in Hela cells and yeast and Hela cell extracts showed similar behaviors. The first experiment included two biological replicates, allowing replicate-based statistical analysis, whereas the second consisted of a single sample–control comparison (see SI, Tables S5 and S6).

Across both experiments, multiple TPR-containing cochaperones known to recognize the conserved MEEVD motif of Hsp90 were identified, including STIP1 (HOP), FKBP4, FKBP5, PPP5C (PP5), PPID (Cyp40),^50^ SGTA,^51^ and STUB1 (CHIP),^52^ despite the use of the yeast CTD as a bait. The consistent recovery of these proteins provides evidence that IsoC687-Gd preserves sequence-specific recognition of MEEVD-binding TPR domains.

Notably, the observed enrichment levels were generally modest, as expected given the highly competitive cellular environment and the presence of abundant endogenous Hsp90, which engages the same pool of TPR-domain proteins. To evaluate specificity, independent of absolute enrichment magnitude or protein ranking, we performed a class-level analysis comparing enrichment distributions of TPR-containing proteins with those of non-TPR proteins (Figure S18 and Tables S7 and S8). In experiment 2, TPR-containing proteins extended further into the high-enrichment regime, whereas in experiment 1, they were predominantly clustered at moderate enrichment values. Importantly, inspection of individual TPR cochaperones revealed that five of them were reproducibly recovered above background across experiments, even when enrichment values were moderate. Together, these results demonstrate that IsoC687-Gd specifically engaged TPR-domain.

These findings provide a molecular basis for the conformational compaction of IsoC observed in cell extracts and in cells, supporting the suggestion that engagement of endogenous TPR-domain partners recapitulates the structural effects of Cpr6 binding observed in vitro.

## Conclusions

In this work, we investigated the conformational space of the intrinsically disordered CTD tail, consisting of the MEEVD motif at its extreme end, and its impact on yHsp90 structure in the apo-state and upon binding of the cochaperone Cpr6. We show that the conformational ensemble of the CTD tail differs markedly between IsoC and FL yHsp90, most likely due to distinct interactions of the tail with the CTD and the adjacent middle domain in the full-length protein. Notably, these differences are abolished upon Cpr6 binding, suggesting that engagement of the MEEVD motif constrains the tail into a common conformational state.

In IsoC, the CTD tail also modulates the folded CTD’s conformation, giving rise to a secondary population near the dimer interface. The presence of this population is consistent with tail–CTD interactions supported by contacts with the folded CTD region that are not accessible in the FL protein. Removal of the tail eliminates this secondary population in IsoC and induces a structural change in the FL CTD, thereby abolishing the differences between IsoC and FL. These observations demonstrate that, in both constructs, the CTD tail contributes to shaping the CTD’s structural properties. Binding of Cpr6 reduces the extent of the secondary CTD population in IsoC, shifting its conformational distribution toward that observed for FL. Importantly, this effect is preserved in yeast and HeLa cell extracts, as well as in HeLa cells, where endogenous TPR-containing partners interact with the IsoC tail.

Together, our results demonstrate that the CTD tail is not merely a flexible appendage that mediates recruitment of TPR-containing cochaperones via the MEEVD motif. Instead, it engages in intramolecular interactions with other Hsp90 domains, thereby modulating CTD structure at the dimer interface. These findings reveal previously underappreciated structural roles of the CTD tail and motivate future studies addressing if and how tail–domain interactions may contribute to the regulation of cochaperone and client binding during the Hsp90 functional cycle.

## Materials and Methods

### Protein Expression and Purification

All FL and IsoC yHsp90 variants were expressed and purified following literature protocols.^17,18^ For IsoC variants, the step of size exclusion chromatography was omitted. Expression and three-step chromatographic purification of Cpr6 was carried out according to published procedures.^53^ The purity of the proteins was confirmed by SDS-PAGE and mass spectrometry.

For NMR measurement, IsoC and IsoCΔ constructs were cloned into pETM11 vector and expressed with an N-terminal His-TEV tag. Proteins were expressed in *Escherichia coli* BL21 DE3 in Minimal M9 media substituted with ^15^N-NH_4_Cl for ^15^N-labeling. Expression was induced with 0.5 mM IPTG and expressed overnight at 20 °C. Cells were lysed using sonication, followed by Ni-NTA affinity purification, dialysis and TEV cleavage overnight, reverse Ni-NTA for tag removal and size exclusion chromatography into NMR Phosphate buffer (20 mM phosphate buffer, 100 mM NaCl, 2 mM DTT, pH 6.8).

### Labeling of Protein with Gd­(III) Spin Label and MSL

Gd­(III) labeling with BrPy-DO3A-Gd­(III) was done according to previous protocols.^17,18^ Briefly, the Gd­(III) label (10 equiv in Milli-Q water, pH 6.5) was added to the reduced mutants with 3,3′,3″-phosphanetriyltripropanoic acid, TCEP (1 equiv in Milli-Q water), the pH was adjusted to 8.5 and the labeling was performed overnight at room temperature (RT). The excess label was removed with Vivaspin 500 μL concentrator with 30 kDa (for FL) or 10 kDa (for IsoC) molecular cutoff via continuous buffer exchange (×5) using the DEER buffer (20 mM Tris·HCl, 20 mM KCl, in D_2_O, pD 7.4). Glycerol-*d*_8_ was added at the end to yield a 20% (v/v) glycerol solution. The protein stock was further diluted as needed.

Nitroxide labeling with 3-maleimido-proxyl (5-MSL, Sigma) was done in 20 mM Tris·HCl buffer, 20 mM KCl, pH = 7.4 where the mutants were incubated with TCEP (5–10 equiv in Milli-Q water) for 30 min, then MSL (5 equiv in Milli-Q water) was added to the mixture without TCEP removal. The pH was adjusted to 7.0 and the labeling was performed for 6 h at RT, and excess of spin label was removed via G-25 spin desalting column using 20 mM Tris·HCl, 20 mM KCl, pH 7.4 buffer.

The degree of labeling was verified by size-exclusion chromatography coupled time-of-flight mass spectrometry (SEC-TOF-MS), showing a quantitative labeling for all variants. Mass spectra were recorded as full MS scans on TOF mass spectrometer (TOF-MS) equipped with an electrospray ionization source in positive polarity (ES+). For the measurements, 10 μL of protein (before and after labeling) were diluted with a 100 mM ammonium acetate buffer (pH ∼ 6.0) to a final volume 50 μL, yielding a final protein concentration of 20–50 μM (as monomer). We used MSL because labeling with the more commonly used spin label, MTSL, led to partial precipitation of the protein.

### Labeling of Protein with a Fluorescent Dye (ATTO488)

Labeling with ATTO488 for observation under a fluorescence microscope was done on the IsoC637 and FL637. The mutants were treated with TCEP (1 equiv in Milli-Q water), before addition of ATTO488 (1.5 equiv in Milli-Q water). The reaction proceeded for 2 h at RT in the dark, and excess of dye was removed using Micro Bio-Spin Size Exclusion Spin Columns and exchanged (×2) with 20 mM Tris·HCl, 20 mM KCl, pH 7.4, yielding 20 μL of ∼200 μM fluorescently labeled proteins.

### Yeast Cell Extract and Sample Preparation

Cell extracts were produced from spheroplasts, which lack polysaccharide-rich cell walls of the yeast cells. Yeast was grown to the desired phase (OD_600_ = 0.5–0.6), yielding approximately 35 × 10^6^ cells in 5 mL of growth medium. The culture was chilled on ice for 5 min, then centrifuged at 3,000 × g for 5 min at 4 °C. The pellet was resuspended in 1 mL of ice-cold spheroplast buffer (1.2 M sorbitol, 20 mM potassium phosphate, 1 mM MgCl_2_, pH 7.4–7.5), zymolyase was added to a final concentration of 0.1 mg/mL, and the mixture was incubated for 30–45 min at 30 °C with gentle rotation. The suspension was gently centrifuged at 2,000 × g for 5 min at 4 °C and the supernatant was discarded. The resulting spheroplasts were gently resuspended in ice-cold lysis buffer (50 mM Tris·HCl, 150 mM NaCl, 0.1% NP-40 and 1:1000 fresh protease inhibitor cocktail), kept on ice for 5 min and centrifuged at 15,000 × g for 10 min at 4 °C. The supernatant, containing the cell extract, was aliquoted and snap-frozen at −80 °C. The Bradford assay yielded a total protein content in the cell extract of 113 mg/mL.

Spin-labeled yHsp90 construct, D_2_O, and glycerol were added to achieve a final extract concentrations 11 mg/mL (10%) and 22 mg/mL (20%). Samples for DEER measurements contained 50 μM Hsp90 (as monomer), 10% or 20% yeast cell extract and 20% glycerol-*d*_8_.

### HeLa Cell Extract Preparation and Sample Preparation

HeLa cell extract was prepared according to published protocol^49^ starting from 42 × 10^6^ cells/mL. The Bradford assay yielded a total protein content of 110 mg/mL in the cell extract. Spin-labeled yHsp90, D_2_O, and glycerol were added to achieve a final sample composition of 10% and 20% CE. Samples for DEER measurements contained 50 μM Hsp90 constructs (as monomer), 10% or 20% HeLa cell extract and 20% glycerol-*d*_8_.

### Cell Delivery by Electroporation

Delivery of Gd-labeled constructs into living HeLa cells was performed according to established protocols, utilizing electroporation as the delivery method.^54^ Specifically, HeLa cells from three plates having ∼80% confluency were collected and washed with electroporation buffer (100 mM sodium phosphate, 5 mM KCl, 15 mM MgCl_2_, 15 mM HEPES, 0.1 mM ATP, 0.1 mM reduced glutathione, pH 7.4). The Gd-labeled variants were added to 200 μM isoC or 100 μM FL final concentration, and the mixture was transferred to 2 mm electroporation cuvette and electroporated twice using a Lonza Nucleofector b system at program B28. After the first electroporation, the cells were gently remixed before the second electroporation. The cells were then transferred to a pretreated collagen plate, avoiding picking cell debris that formed as a result of the electroporation procedure. The collagen plate containing the transfected cells was incubated in a CO_2_-rich chamber at 32 °C for 4 h; during this time, cells were inspected under a microscope to ensure they maintained their shape and did not undergo apoptosis. After 4 h the cells were detached from the plate with trypsin digestion, washed thrice with PBS buffer (100 mM sodium phosphate, pH 7.4) to remove dead cells and noninternalized protein, and incubated for 5 min in deuterated PBS buffer containing 20% glycerol-*d*_8_. Cells were loaded into EPR capillaries (0.6 ID/0.84 OD mm or 0.8 ID/1.00 OD mm), the capillaries were centrifuged at 1,500 × g for 30 min so that the cell pellet was collected at the bottom of the capillary, the upper part (containing just buffer) was cut and the sample was frozen slowly in isopropanol rack at −80 °C.

### Sample Preparation for Pull-Down Experiments

Samples for mapping protein–protein interactions by affinity pull-down followed by tryptic digestion and mass spectrometry were prepared as follows. The bait was IsoC687-Gd, which carries a His_6_ tag and was used in a Ni-NTA affinity purification assay.

HeLa cell lysate (110 mg/mL, 1 μL) was mixed with either 4 μL Milli-Q water–water (control, C) or with 4 μL IsoC637-Gd (sample, S), resulting in a final IsoC687-Gd concentration of 50 μM in S. Ni-NTA agarose beads were equilibrated (6 × 100 μL) with binding buffer (50 mM Tris·HCl, 150 mM NaCl, 10 mM imidazole, pH 8.0). For each pull down reaction, 20 μL of equilibrated beads were combined with 5 μL of either C or S mixture and incubated for 1 h at room temperature with gentle mixing to allow binding. Two independent experiments were carried out, experiment 1 with duplicates and experiment 2 as a single sample.

Following incubation, bound proteins were eluted (3 × 50 μL) by addition of elution buffer (50 mM Tris·HCl, 300 mM NaCl, 250 mM imidazole, pH 8.0). The elutions from each replicate were combined and used for subsequent mass spectrometry analysis.

#### Liquid Chromatography Mass Spectrometry

ULC/MS grade solvents were used for all chromatographic steps. Each sample was loaded using split-less nanoultra performance liquid chromatography (10 kpsi nanoACQUITY; Waters, Milford, MA, USA). The mobile phase was as follows: (A) H_2_O + 0.1% formic acid and (B) acetonitrile + 0.1% formic acid. Desalting of the samples was performed online using a reversed-phase Symmetry C18 trapping column (180 μm internal diameter, 20 mm length, 5 μm particle size; Waters). The peptides were then separated using a T3 HSS nanocolumn (75 μm internal diameter, 250 mm length, 1.8 μm particle size; Waters) at 0.35 μL/min. Peptides were eluted from the column into the mass spectrometer using the following gradient: 4 to 30% B in 50 min, 30 to 90% B in 5 min, maintained at 90% for 5 min and then back to initial conditions.

The nanoUPLC was coupled online through a nanoESI emitter (10 μm tip; New Objective; Woburn, MA, USA) to Q Exactive Plus mass spectrometer (Thermo Scientific). Data were acquired in data-dependent acquisition (DDA) mode, using a Top10 method. MS1 resolution was set to 70,000 (at 200 *m*/*z*), mass range of 375–1,500 *m*/*z*, AGC of 1 × 10^6^ and maximum injection time was set to 60 ms. MS2 was performed by isolation with the quadrupole, window of 1.7 *m*/*z*, 27 NCE, 17,500 resolution, AGC target of 1e5, maximum injection time of 60 ms and dynamic exclusion of 25 s.

#### Data Processing

Raw data were analyzed using the MaxQuant software suite 1.6.6.0 ^100^ with the Andromeda search engine. The higher-energy collisional dissociation (HCD) MS/MS spectra were searched against an *in silico* tryptic digest of human proteins from the UniProt/Swiss-Prot sequence database (v. Jan-2021), the amino acid sequence of IsoC, including common contaminant proteins. All MS/MS spectra were searched with the following MaxQuant parameters: acetyl (protein N-terminus), M oxidation and NQ-deamidation. Cysteine carbamidomethylation was set as fixed modification. Max 2 missed cleavages; and precursors were initially matched to 4.5 ppm tolerance and 20 ppm for fragment spectra. Peptide spectrum matches and proteins were automatically filtered to a 1% false discovery rate based on Andromeda score, peptide length, and individual peptide mass errors.

Proteins were identified and quantified based on at least one unique peptide and based on the label-free quantification (LFQ^55^) values reported by MaxQuant.

Two independent MaxQuant data sets were analyzed separately using identical filtering criteria to assess reproducibility. In each data set, proteins annotated as potential contaminants were removed. Only proteins identified with at least two unique peptides and two MS/MS spectra were retained. Enrichment was assessed using LFQ intensity ratios (Sample vs Control) and associated *Q*-values.

For the data set, which contained a single sample and control measurement, enrichment was calculated as the log_2_ fold change of LFQ intensities (sample versus control). For the data set, which contained two replicates per condition (C1/C3 and S1/S2), LFQ intensities were log_2_-transformed and averaged per condition, and enrichment was calculated as the difference between mean log_2_ sample and mean log_2_ control intensities. Statistical significance in data set 1 was assessed using a Welch’s *t* test on log_2_-transformed replicate intensities, followed by Benjamini–Hochberg false discovery rate correction.

Protein groups were annotated based on gene names and protein descriptions and classified into functional categories, including TPR cochaperones, mitochondrial/metabolic proteins, cytoskeletal/structural proteins, ribosomal/translation proteins, and other potential clients or background binders. Proteins containing TPR domains known to bind the conserved MEEVD motif of the Hsp90 C-terminal domain were manually curated based on published literature and annotated as bona fide IsoC binders. For downstream interpretation, TPR proteins were collapsed to the gene level by retaining the maximum observed log_2_ fold enrichment per gene. Comparative analyses between data sets were performed at the gene level to assess reproducibility and specificity of Hsp90 C-terminal domain interactions.

### Surface Plasmon Resonance (SPR)

SPR measurements were conducted using Biacore 8K (Cytiva). Hsp90 samples were immobilized onto a Series S CM5 chip, using Amine Coupling kit (Cytiva), with 50 mM PBS as the running buffer. Final immobilized response units (RUs) of FL and IsoC samples were ∼700 RUs and ∼4000 RUs, respectively. The binding of Cpr6 to immobilized Hsp90 constructs was analyzed by injecting 2-fold serial dilutions of Cpr6 at a flow rate of 30 μL/min for 120 s, using 20 mM Tris·HCl, 20 mM KCl, 0.05% Tween20 as the running buffer at 25 °C. For each sample the affinity of the interaction was determined with a Langmuir binding model: $R\left(c\right)=R_{0}+R_{\max}\cdot \frac{c}{K_{d}+c}$ , where *R*(*c*) is steady-state SPR response at ligand concentration *c*, *R*_0_ is baseline of non–specific signal or instrumental offset measured in the absence of ligand, *R*_max_ is the maximum specific binding signal attained when all sites are saturated, and *K*_d_ is the equilibrium dissociation constant. For competition experiments, measurements were conducted using Biacore S200 (Cytiva), where Hsp90 samples were immobilized as mentioned above to final RU values of ∼750 RUs. Competition experiments were done by injecting serial dilutions of Hsp90 samples mixed with fixed 2.5 μM of Cpr6, for 120 s, at a flow rate of 30 μL/min, at 25 °C, using 20 mM Tris·HCl, 20 mM KCl, 0.05% v/v Tween20 as running buffer. The analysis of the competition data was done as described in the literature^34^ and the SI.

### Microscale Thermophoresis (MST)

MST experiments were performed at 25 °C on a micro-MST Monolith instrument (NanoTemper Technologies). Each mutant was labeled with DyLight 650 (ThermoFisher) and incubated for 60 min in PBS buffer (10 mM Na_2_HPO_4_, 2 mM KH_2_PO4, 137 mM NaCl, 2.5 mM KCl, at pH 7.8) in the dark. Free dye was then removed by PD-10 column. Labeling efficiencies were 50–100% as measured by Carey 60 UV/vis spectrophotometer (Agilent Technologies). A 16-step 1:1 (v/v) serial dilution of the unlabeled protein (titrant) in the exchange buffer (20 mM Tris·HCl, pH 7.5, 20 mM KCl, 0.05% Tween-20) was prepared; then the labeled protein (target) was added to each sample in the same amount, resulting in an additional 1:1 dilution.^56,57^ The samples were centrifuged at 21,000 × g at 4 °C for 10 min to remove any precipitates and were then loaded into Monolith capillaries (NanoTemper Technologies, MO-K022). The concentration of the target was kept at 20 nM throughout the experiments, whereas the concentration of the titrant ranged from 84 μM up to 100 μM. Experiments were run at 100% fluorescence power and MST powers: for isoC637 and FL637–medium power, for isoCΔ−low power, for FLΔ−high power. The data for samples FL637, FLΔ and isoC637 were fitted as described earlier,^19^ while the data for isoCΔ was fitted with the Morrison (tight-binding) equation^34^

$f\left(c\right)=F_{\mathrm{target}}+\left(F_{\mathrm{complex}}-F_{\mathrm{target}}\right)\times \frac{c+c_{\mathrm{target}}+K_{d}-\sqrt{{\left(c+c_{\mathrm{target}}+K_{d}\right)}^{2}-4\cdot c\cdot c_{\mathrm{target}}}}{2c_{\mathrm{target}}}$ , where *f*(*c*) is the fraction bound at a given ligand concentration *c*; *F*_target_ is the normalized signal of the target alone; *F*_complex_ is the normalized signal of the complex; *K*_d_ is the dissociation constant; and *c*_target_ is the final concentration of target in the assay.

### Nanoscale Differential Scanning Fluorimetry (NanoDSF)

NanoDSF measurements were performed using a Prometheus Panta instrument (NanoTemper Technologies). Protein samples in 50 mM PBS buffer were loaded into standard-grade capillaries (NanoTemper Technologies, Cat. No. PR-C002). The temperature increased from 25 °C to 95 °C at a heating rate of 1 °C/min. Intrinsic protein fluorescence was monitored at 330 nm and 350 nm. The melting temperature (*T*_m_) was determined by calculating the first derivative of the fluorescence ratio (*F*_350_/*F*_330_) as a function of temperature.

### EPR Measurements and Data Analysis

Continuous wave (CW) X-band (9.5 GHz) EPR spectra were carried out on a Bruker Magnettech ESR5000 spectrometer with a modulation frequency of 100 kHz, modulation amplitude of 0.1 mT, and a microwave (MW) power of 1 mW. Samples were placed into quartz capillaries of 1 mm outer diameter and 0.8 mm inner diameter filled to a height of 2.5 cm. Both ends of the capillaries were sealed with critoseal. All simulations were carried out with SimLabel,^58^ a Matlab GUI build on the Easyspin toolbox.^59^

All pulsed EPR measurements were carried out at 10 K on a home-built W-band spectrometer (94.9 GHz).^60,61^ DEER time traces were recorded with the 4-pulse DEER sequence (π/2­(ν_obs_) – τ_1_ – π­(ν_obs_) – (τ_1_ + *t*) – π­(ν_pump_) – (τ_2_ – *t*) – π­(ν_obs_) – τ_2_ – echo)^62^ using a chirp pump pulse^63–65^ and monitoring the echo intensity with increasing *t*. A four-step phase cycling was applied to remove unwanted echoes. The maximum of the Gd­(III) signal was set to 95.00 GHz. The microwave pulse lengths at the observe frequency (94.825 GHz) were π/2­(ν_obs_) = 15 ns and π­(ν_obs_) = 30 ns, and the repetition time was 200 μs. The interpulse delays were τ_1_ = 600 μs, τ_2_ = 4.5/5 or 2.5 μs when the concentration was too low to measure the sample in a reasonable time. The step of *t* was 20 ns the starting *t* was −200 ns. The length of the chirp pulse was 128 ns, covering a frequency range of 150 MHz (94.925–95.075 GHz), while for in-cell measurements, the chirp pulse was 128 ns and the pump frequency range was 94.9–95.05 GHz.

### DEER Data Analysis

The DEER data were transformed into distance distributions using Tikhonov regularization within DeerAnalysis2022 software^37^ ran via Matlab2022b and the background was fit with a homogeneous three-dimensional distribution. The DEER measurements with various amounts of Cpr6 and cell extracts and in cell were analyzed by using GLADDvu, a MATLAB software developed by the Hustedt Lab for globally fitting multiple DEER data sets.^1^

IsoC637*-Gd + Cpr6*: The six data sets were analyzed by using two Gaussians (r_01_, σ_r01_ and r_02_, σ_r02_). r_0_ and σ_r0_ were determined by fitting the IsoC637 without the presence of Cpr6. The best-fit values were fixed during the global analysis and were linked across all six data sets, while the amplitudes of the components and the modulation depths varied.

*FL637-Gd + Cpr6*: The two data sets were analyzed by using one Gaussian. r_0_ and σ_r0_ for the two data sets were fit and linked to each other during the global analysis.

*IsoC637-Gd in 10% and 20% Yeast cell extract (CE)*: The three data sets were analyzed by using two Gaussians (r_01_, σ_r01_ and r_02_, σ_r02_). r_0_ and σ_r0_ were determined by fitting the IsoC637 data without the presence of CE. The best-fit values were fixed during the global analysis and were linked across all three data sets while the amplitudes of the components and the modulation depths varied.

*FL637-Gd in 10% and 20% Yeast CE*: The three data sets were analyzed by using one Gaussian. r_0_ and σ_r0_ for the two data sets were fit and r_0_ linked during the global analysis.

*IsoC637-Gd in 10% and 20% Hela CE and in Hela cells*: The four data sets were analyzed by using two Gaussians (r_01_, σ_r01_ and r_02_, σ_r02_). r_0_ and σ_r0_ were determined by fitting the IsoC637 without the presence CE. The best-fit values were fixed during the global analysis. The two r_0_ and σ_r02_ were linked across all four data sets while the amplitudes of the components and the modulation depths varied.

*FL637-Gd + 10% and 20% Hela CE and in Hela Cells*: The four data sets were analyzed by using one Gaussian. r_0_ and σ_r0_ for the two data sets were fit and r_0_ linked during the global analysis.

*IsoC/FL560-Gd + Cpr6 and + Yeast CE*: The data sets were analyzed using one Gaussian, the r_0_s were determined by fitting the isoC/FL560-Gd without the presence of Cpr6/CE. The best-fit values were fixed and r_0_ linked during the global analysis.

*IsoC/FL627-Gd + Cpr6 and + Yeast CE*: The data sets were analyzed using one Gaussian, the r_0_s were determined by fitting the isoC/FL 627-Gd without the presence of Cpr6/CE. The best-fit values were fixed, and r_0_ was linked during the global analysis.

### NMR Measurements

NMR measurements were performed on a 1.2 GHz Bruker spectrometer equipped with a cryogenically cooled TCI probehead. ^1^H,^15^N HSQC experiments were performed at 298 K with 500 μM protein in 20 mM phosphate buffer, 100 mM NaCl, 2 mM DTT, pH 6.8, 7% D_2_O. Data was processed using NMRPipe^66^ and analyzed using CCPNMR.^67^ The chemical shift assignment of the CTD was previously reported.^66^

### AlphaFold Structure Prediction

Prediction of the structure of the complexes of yHsp90 (FL and IsoC) with one or two Cpr6 molecules was done using AlphaFold server,^68^ an online platform which uses AlphaFold 3, a Google DeepMind and Isomorphic Laboratories collaboration model. For this, the FASTA sequences of relevant proteins were added using the “Add entity” function. AlphaFold generated five predicted structural models per submission, and among those, model zero (model_0.cif) generally had the highest confidence with a Predicted Local Distance Difference Test (pLDDT) score >90.^69^ The generated structures (.cif files) were then visualized using UCSF ChimeraX.^70^ Additionally, the 5 structures were loaded to MtsslWizard software to anchor the GdDO3A label on each of them followed by measurement of the interspin distances.^40^

### Homology between Human and Yeast Hsp90

Protein sequence homology was assessed using BLAST alignment with UniProt entries P46598 (yeast Hsp90; *Candida albicans*, strain SC5314/ATCC MYA-2876) and P07900 (human Hsp90; *Homo sapiens*).

## Supplementary Material











## Notes

Sequences of protein variants and corresponding SDS-PAGE and mass spectra, SPR measurements, additional DEER data on FL and IsoC variants labeled at the CTD tail, AlphaFold structures and predicted distance distributions, additional DEER data on FL and IsoC variants labeled at the CTD, NMR measurements, DSF and MST measurements, thermodynamic model, DEER measurements in cells and cell extracts, pull-down experiments and corresponding tables (PDF)
B2RPK0, P09429, and P23497 (Table S5) (XLSX)
AGP1 and Q58FG1 (Table S6) (XLSX)
Q9BWD1 (Table S7) (XLSX)
Q96QA5 (Table S8) (XLSX)

## ○

Department of Chemistry, University of Cambridge, Cambridge CB2 1EW, UK
