# Supplementary material for: Beyond a Passive Tether: Structural Insights into the Disordered Tail of Hsp90
Source: J Am Chem Soc. 2026 Apr 9;148(15):16469–81. doi: 10.1021/jacs.6c04189 (PMC13107436; doi:10.1021/jacs.6c04189)
Supplement: Supplementary file 1 [file ja6c04189_si_001.pdf]

Supporting Information

Beyond a Passive Tether: Structural Insights into the Disordered Tail of Hsp90

Elena Edinach,[1&] Angeliki Giannouli,[1,2&] Arina Dalaloyan,[1] Maria Oranges,[1] Debasis Banik,[1,#] Elian Hadas Yardeni,[3] Annika Elimelech,[4] Michael Sattler,[4] Emmanouil Ntermanakis,[2] Xun-Cheng Su,[5] Daniella Goldfarb[1,*]

[1]Department of Chemical and Biological Physics, Weizmann Institute of Science, Rehovot, 7610001, Israel

[2]Department of Chemistry, University of Crete, Heraklion, 70013, Greece

[3]Protein Analysis Unit, Department of Life Sciences Core Facilities, Weizmann Institute of Science, Rehovot, 7610001, Israel

[4]Institute of Structural Biology, Molecular Targets and Therapeutics Center, Helmholtz Munich, Neuherberg, 85764, Germany and Bavarian NMR Center and Department of Bioscience, TUM School of Natural Sciences, Technical University of Munich, Garching, 85748, Germany

[5]State Key Laboratory of Elemento-Organic Chemistry, College of Chemistry, Nankai University, Tianjin, 300071, China

Contents

1. Sequences of FL, isoC, FLΔ, and IsoCΔ S2
2. SDS-PAGE and mass spectra of the variants studied in this work S3
3. SPR measurements S6
4. Additional DEER data on FL and IsoC variants labeled at the CTD tail S8
5. AlphaFold structures and predicted distance distributions S9
7. Additional DEER data on FL and IsoC variants labeled at the CTD S12
8. NMR measurements S13
9. DSF and MST measurements S14
10. Thermodynamic model S15
11. DEER measurements in cells and cell extracts S16
12. Pull-down experiments S17

1. Sequences of **FL, isoC, FLΔ, and IsoCΔ**

FL, D560, K627, K637, A687, A701 are marked in red
MGSSHHHHHHSSGASETFEFQAEITQLMSLIINTVYSNKEIFLRELISNASDALDKIRYKSL
SDPKQLETEPDLFIRITPKPEQKVLEIRDSGIGMTKAELINNLGTIAKSGTKAFMEALSAGA
DVSMIGQFGVGFYSLFLVADRVQVISKSNDDEQYIWESNAGGSFTVTLDEVNERIGRGTIL
RLFLKDDQLEYLEEKRIKEVIKRHSEFVAYPIQLVVTKEVEKEVPIPEEEKKDEEKKDEEK
KDEDDKKPKLEEVDEEEEKKPKTKKVKEEVQEIEELNKTKPLWTRNPSDITQEEYNAFY
KSISNDWEDPLYVKHFSVEGQLEFRAILFIPKRAPFDLFESKKKKNNIKLYVRRVFITDEAE
DLIPEWLSFVKGVVDSEDLPLNLSREMLQQNKIMKVIRKNIVKKLIEAFNEIAEDSEQFEK
FYSAFSKNIKLGVHEDTQNRAALAKLLRYNSTKSVDELTSLTDYVTRMPEHQKNIYYITG
ESLKAVEKSPFLDALKAKNFEVLFLTDPIDEYAFTQLKEFEGKTLVDITKDFELEETDEEKA
EREKEIKEYEPLTKALKEILGDQVEKVVVSYKLLDAPAAIRTGQFGWSANMERIMKAQA
LRDSSMSSYMSSKKTFEISPKSPIIKELKKRVDEGGAQDKTVKDLTKLLYETALLTSGFSLD
EPTSFASRINRLISLGLNIDEDEETETAPEASTAAPVEEVPADTEMEEVD

FLΔ, K637 is marked in red
MGSSHHHHHHSSGASETFEFQAEITQLMSLIINTVYSNKEIFLRELISNASDALDKIRYKSL
SDPKQLETEPDLFIRITPKPEQKVLEIRDSGIGMTKAELINNLGTIAKSGTKAFMEALSAGA
DVSMIGQFGVGFYSLFLVADRVQVISKSNDDEQYIWESNAGGSFTVTLDEVNERIGRGTIL
RLFLKDDQLEYLEEKRIKEVIKRHSEFVAYPIQLVVTKEVEKEVPIPEEEKKDEEKKDEEK
KDEDDKKPKLEEVDEEEEKKPKTKKVKEEVQEIEELNKTKPLWTRNPSDITQEEYNAFY
KSISNDWEDPLYVKHFSVEGQLEFRAILFIPKRAPFDLFESKKKKNNIKLYVRRVFITDEAE
DLIPEWLSFVKGVVDSEDLPLNLSREMLQQNKIMKVIRKNIVKKLIEAFNEIAEDSEQFEK
FYSAFSKNIKLGVHEDTQNRAALAKLLRYNSTKSVDELTSLTDYVTRMPEHQKNIYYITG
ESLKAVEKSPFLDALKAKNFEVLFLTDPIDEYAFTQLKEFEGKTLVDITKDFELEETDEEKA
EREKEIKEYEPLTKALKEILGDQVEKVVVSYKLLDAPAAIRTGQFGWSANMERIMKAQA
LRDSSMSSYMSSKKTFEISPKSPIIKELKKRVDEGGAQDKTVKDLTKLLYETALLTSGFSLD
EPTSFASRINRLISLGLNI

IsoC, D560, K627, K637, A687, A701 are marked in red
MGSSHHHHHHSSGTKDFELEETDEEKAEREKEIKEYEPLTKALKEILGDQVEKVVVSYKL
LDAPAAIRTGQFGWSANMERIMKAQALRDSSMSSYMSSKKTFEISPKSPIIKELKKRVDEG
GAQDKTVKDLTKLLYETALLTSGFSLDEPTSFASRINRLISLGLNIDEDEETETAPEASTAAP
VEEVPADTEMEEVD

IsoCΔ, K637 is marked in red
MGSSHHHHHHSSGTKDFELEETDEEKAEREKEIKEYEPLTKALKEILGDQVEKVVVSYKL
LDAPAAIRTGQFGWSANMERIMKAQALRDSSMSSYMSSKKTFEISPKSPIIKELKKRVDEG
GAQDKTVKDLTKLLYETALLTSGFSLDEPTSFASRINRLISLGLNI

Cpr6
MGSSHHHHHHRPKTFFDISIGGKPQGRIVFELYNDIVPKTAENFLKLCEGNAGMAKTKPD
VPLSYKGSIFHRVIKDFMCQFGDFTNFNGTGGESIYDEKFEDENFTVKHDKPFLLSMANA
GPNTNGSQAFITCVPTPHLDGKHVVFGEVIQGKRIVRLIENQQCDQENNKPLRDVKIDDC
GVLPDDYQVPENAEATPTDEYGDNYEDVLKQDEKVDLKNFDTVLKAIETVKNIGTEQFK

KQNYSVALEKYVKCDKFLKEYFPEDLEKEQIEKINQLKVSIPLNIAICALKLKDYKQVLVA SSEVLYAEAADEKAKAKALYRRGLAYYHVNDTDMALNDLEMATTFQPNDAAILKAIHN TKLKRKQQNEKAKKSLSKMFS

The underlined sequence is the His_6-tag.

2. SDS-PAGE and mass spectra of the variants studied in this work

Table S1. List of the Hsp90 variants studied.

Protein	Variant	Spin label
isoC	D560C	Gd-DO3A
isoC	K627C	Gd-DO3A
IsoC	K637C	Gd-DO3A
IsoC	A687C	Gd-DO3A, MSL
isoC	A701C	Gd-DO3A, MSL
IsoCΔ	K637C	Gd-DO3A
FL	D560C	Gd-DO3A
FL	K627C	Gd-DO3A
FL	K637C	Gd-DO3A
FL	A687C	Gd-DO3A, MSL
FL	A701C	Gd-DO3A, MSL
FlΔ	K637C	Gd-DO3A
Cpr6	NA	NA

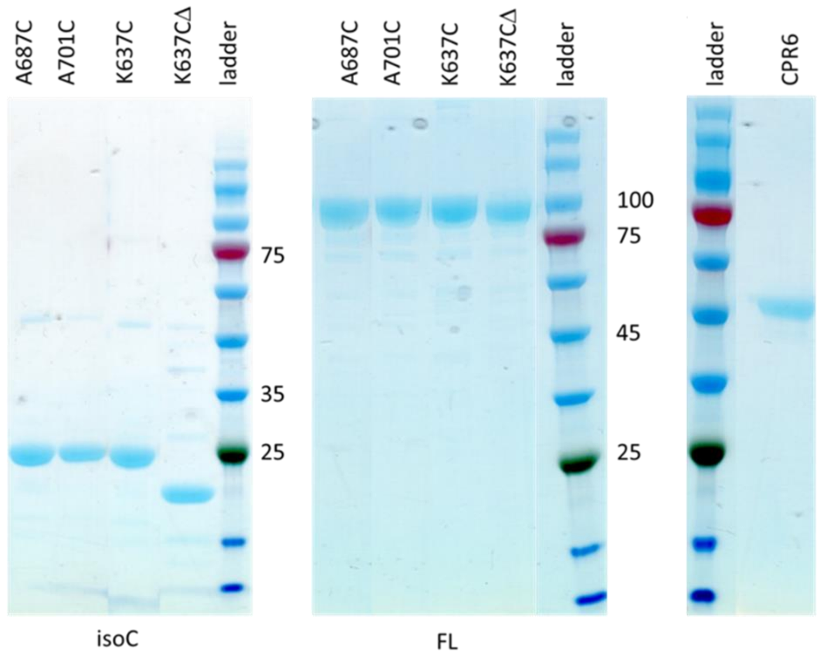

Figure S1. SDS-PAGE analysis of variants A687C and A701C, K637C and K637CΔ IsoC and FL and of Cpr6. Data for the other variants have been reported previously.[1]

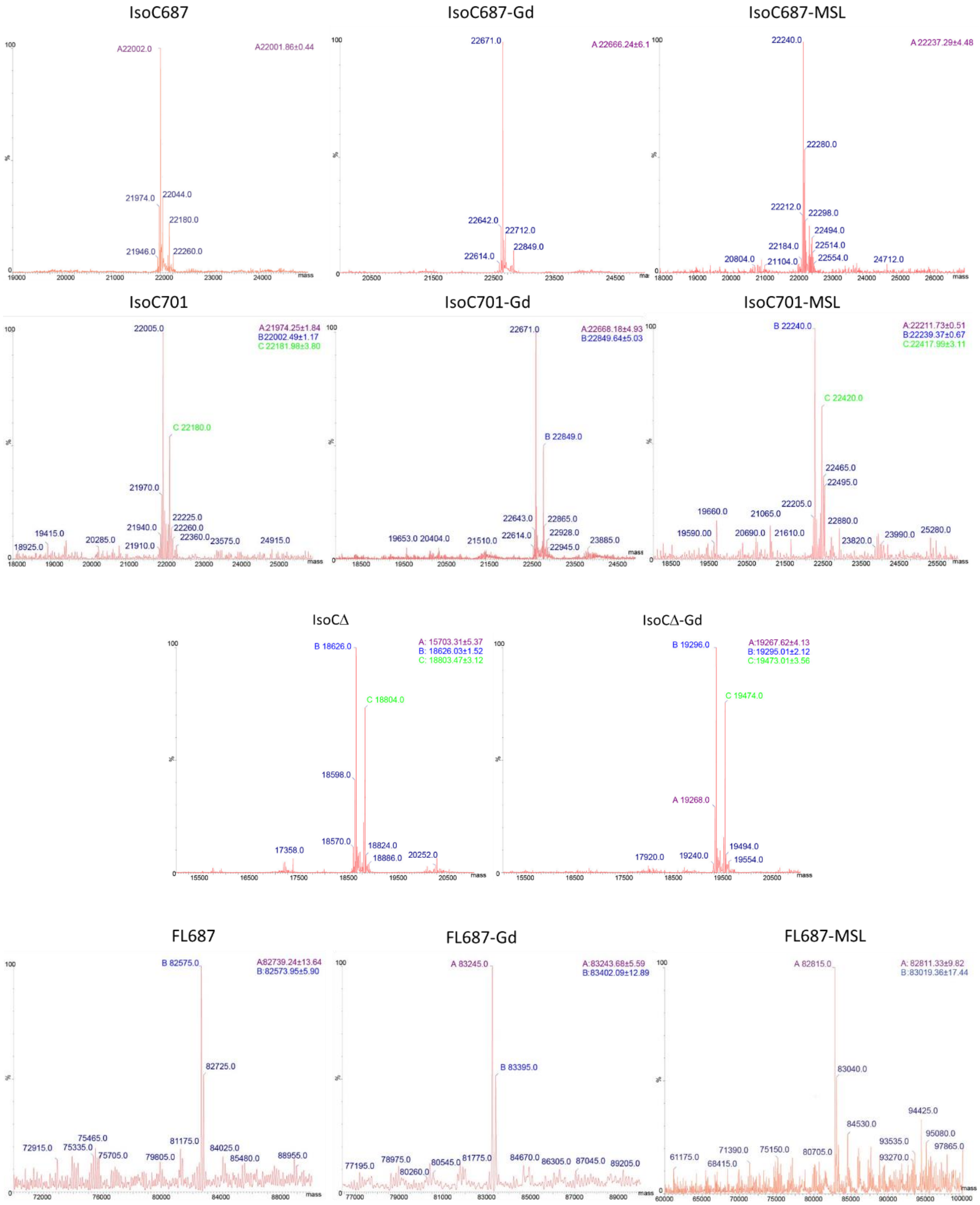
IsoC687
A22002.0
A22001.86±0.44
21974.0
22044.0
22180.0
21946.0
22260.0
IsoC687-Gd
22671.0
A 22666.24±6.1
22642.0
22712.0
22614.0
22849.0
IsoC687-MSL
22240.0
A 22237.29±4.48
22280.0
22212.0
22298.0
22494.0
22184.0
22514.0
20804.0
21104.0
22554.0
24712.0
IsoC701
22005.0
A:21974.25±1.84
B:22002.49±1.17
C:22181.98±3.80
C 22180.0
21970.0
21940.0
22225.0
22260.0
22360.0
19415.0
20285.0
21910.0
23575.0
24915.0
18925.0
IsoC701-Gd
22671.0
A:22668.18±4.93
B:22849.64±5.03
B 22849.0
22643.0
22865.0
22614.0
22928.0
22945.0
19653.0
20404.0
21510.0
23885.0
IsoC701-MSL
B 22240.0
A:22211.73±0.51
B:22239.37±0.67
C:22417.99±3.11
C 22420.0
22465.0
22495.0
22205.0
19660.0
21065.0
19590.00
20690.0
21610.0
22880.0
23820.0
23990.0
25280.0
IsoCΔ
B 18626.0
A: 15703.31±5.37
B: 18626.03±1.52
C: 18803.47±3.12
C 18804.0
18598.0
18570.0
17358.0
18824.0
18886.0
20252.0
IsoCΔ-Gd
B 19296.0
A:19267.62±4.13
B:19295.01±2.12
C:19473.01±3.56
C 19474.0
A 19268.0
17920.0
19240.0
19494.0
19554.0
FL687
B 82575.0
A:82739.24±13.64
B:82573.95±5.90
82725.0
72915.0
75335.0
75465.0
75705.0
79805.0
81175.0
84025.0
85480.0
88955.0
FL687-Gd
A 83245.0
A:83243.68±5.59
B:83402.09±12.89
B 83395.0
77195.0
78975.0
80260.0
80545.0
81775.0
84670.0
86305.0
87045.0
89205.0
FL687-MSL
A 82815.0
A: 82811.33±9.82
B: 83019.36±17.44
83040.0
84530.0
94425.0
95080.0
93535.0
97865.0
61175.0
68415.0
71390.0
75150.0
80705.0
93270.0
mass
%
100
0

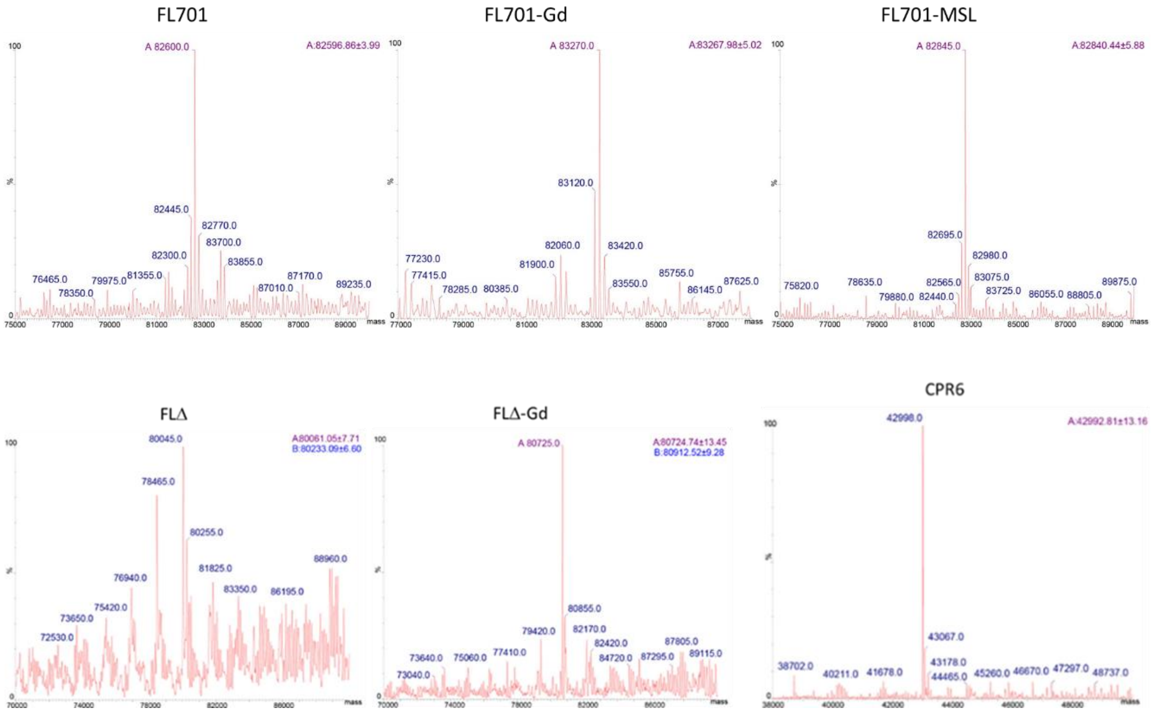

Figure S2. Mass spectra of variants A687C and A701C of IsoC and FL before and after labeling, as well as of Cpr6.

3. SPR measurements

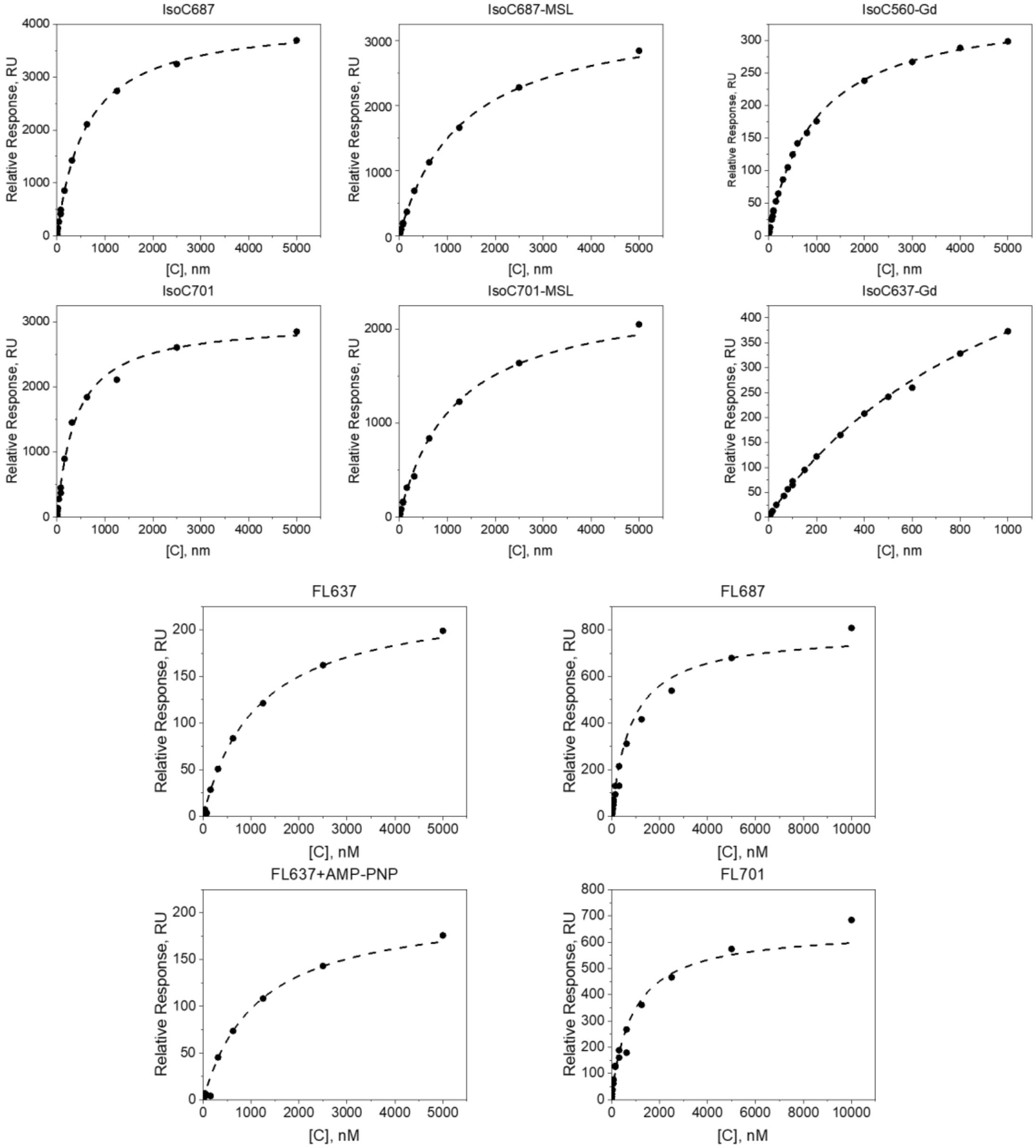

Figure S3A. Direct SPR data of the various variants studied where [C] is the concentration of Cpr6. The dashed lines are the fit to the Langmuir binding model and the derived K_d values are given in **Table S2**.

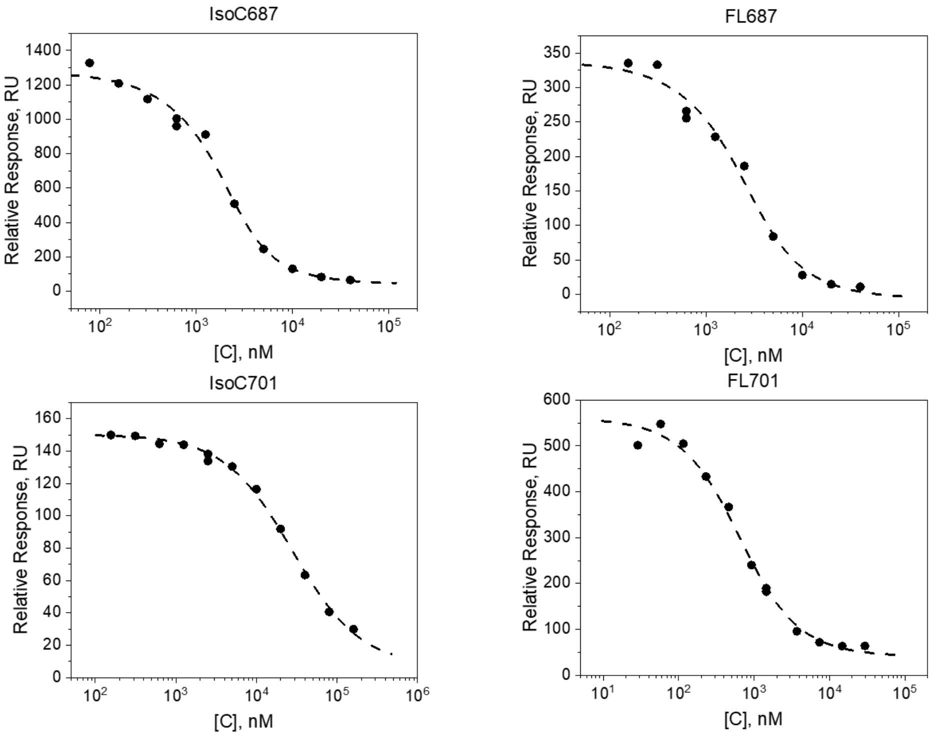

Figure S3B. Competition SPR data of the various variants where [C] is the concentration of the Hsp90 added. The dashed lines are the fit to Morrison (tight-binding) model[2] and the derived K_d values are given in **Table S2**.

Table S2. K_d values of Cpr6 binding with some of the Hsp90 variants determined from SPR measurements.

**Sample**	**K_d / μM**	**Sample**	**K_d / μM**
IsoC687	0.59 ± 0.05<br>0.67 ± 0.43*	FL687	1.07 ± 0.46<br>1.19 ± 0.97*
IsoC687-MSL	1.32 ± 0.14	FL637	1.13 ± 0.24
IsoC701	0.39± 0.07<br>0.27 ± 0.21*	FL637+AMP-PNP	0.91 ± 0.44
IsoC701-MSL	1.16 ± 0.2	FL701	0.09 ± 0.03<br>0.52 ± 0.17*
IsoC637-Gd	1.13± 0.21	IsoC560-Gd	0.97 ± 0.06

*Values determined from competition measurements.

4. Additional DEER data on FL and IsoC variants labeled at the CTD tail

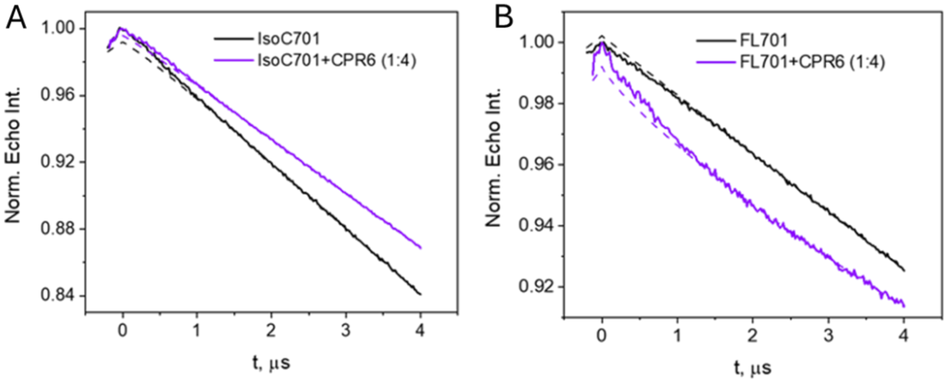

Figure S4. Primary DEER data of (A) IsoC701-Gd and (B) FL701-Gd with and without Cpr6 with the background correction function in dashed lines.

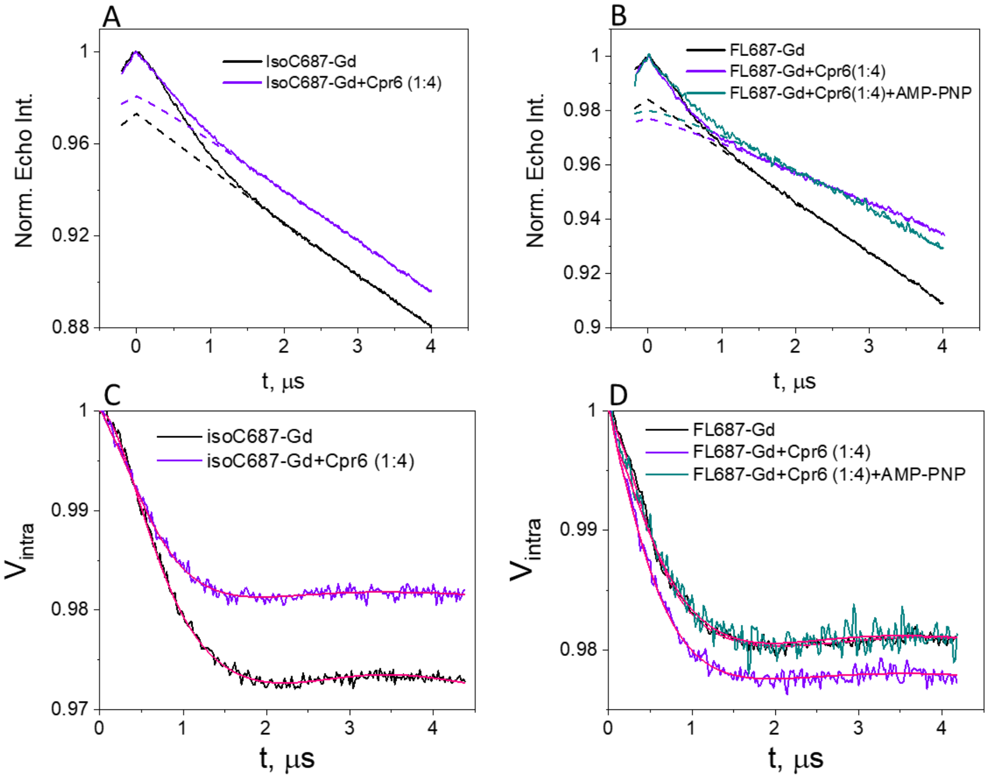

Figure S5. Primary DEER data of (A) IsoC687-Gd and (B) FL687-Gd with and without Cpr6 with the background correction function in dashed lines. Background-corrected DEER data are in C, D, respectively, with the fit in red color using Tikhonov regularization.

5. AlphaFold structures and predicted distance distributions

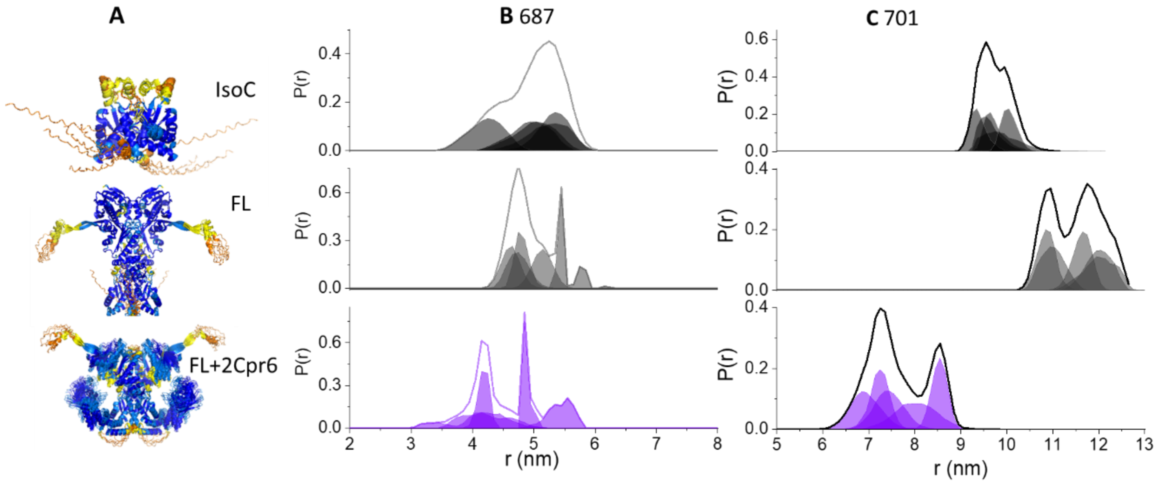

Figure S6. A. AlphaFold structure predictions of the yHsp90 variants. All five output structures are overlaid, and the color codes are: Dark blue = very high confidence (pLDDT > 90), light blue / cyan = confident (pLDDT 70–90), yellow = low confidence (pLDDT 50–70), orange = very low confidence (pLDDT < 50). For apo FL, the model predicts a closed Hsp90 conformation, and not the open conformation which is dominant state in the absence of nucleotides (B) Superposition of the predicted distance distribution between two Gd(III) spin labels attached at position 687 in each of the 5 predicted structures shown in A and their sum (solid line), calculated with MtsslWizard[3]. (C) same as B for position 701. The area of each individual distance distribution was normalized to 1.

6. CW-EPR spectra and simulations

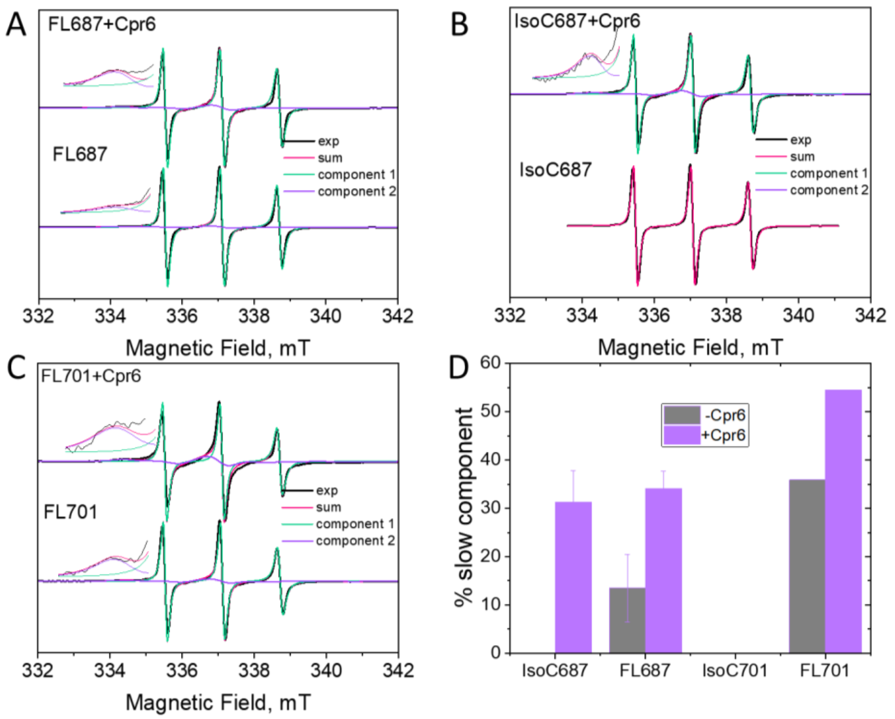

Figure S7. CW-EPR spectra with and without Cpr6 and their simulations for (A) FL687-MSL, (B) IsoC687-MSL and (C) FL701-MSL. In D is the change in the % contribution of the slow-motion component upon Cpr6 binding. The inserts are zooms on the spectral features of the slow-motion component, not expanded to the same scale. The simulation parameters are given in **Table S3**.

Table S3. Parameters used in the EPR simulations for components 1 (fast) and 2 (slow). The top and bottom numbers in the linewidth (lw) column correspond to the Gaussian and Lorentzian broadening, respectively. The principal values of the hyperfine interactions are given under A.

sample	τ(1) / ns	τ(2) / ns	lw(1) / mT	lw(2) / mT	% A(2)	A(1) / mT	A(2) / mT	g(1)	g(2)
FL687	0.12	8.7	0, 0.18	0.3, 0.08	13.5± 7[a]	0.65, 0.65, 3.48	0.67, 0.67, 3.63	2.0091, 2.006, 2.002	2.0091, 2.006, 2.0022
FL687 +Cpr6	0.13 ±0.2	9.5± 0.5	0, 0.17± 0.01	0.25, 0.08	34.1± 3.6	0.65, 0.65, 3.48	0.67, 0.67, 3.63	2.0091, 2.006, 2.002	2.0091, 2.006, 2.0022
IsoC687	0.15		0, 0.17		0	0.65, 0.65, 3.48	0.67, 0.67, 3.63	2.0091, 2.006, 2.002	2.0091, 2.006, 2.0022
IsoC687 +Cpr6	0.15	8.5± 0.25	0, 0.17	0.25, 0.08	31.3± 6.5	0.65, 0.65,	0.67, 0.67,	2.0091, 2.006,	2.0091, 2.006,

						3.48	3.63	2.002	2.0022
FL701	0.2	9	0, 0.16	0.25, 0.08	35.9	0.65, 0.65, 3.49	0.67, 0.67, 3.61	2.0093, 2.0065, 2.0025	2.0095, 2.0064, 2.0022
FL701 +Cpr6	0.2	7.94	0, 0.16	0.25, 0.08	54.5	0.65, 0.65, 3.49	0.67, 0.67, 3.63	2.0093, 2.0065, 2.0025	2.0097, 2.0065, 2.0025
IsoC701	0.15		0, 0.16		0	0.65, 0.65, 3.49		2.0093, 2.0065, 2.0025	
IsoC701 +Cpr6	0.07		0, 0.16		0	0.65, 0.65, 3.49		2.0093, 2.0065, 2.0025	

[a]Error was determined from duplicates.

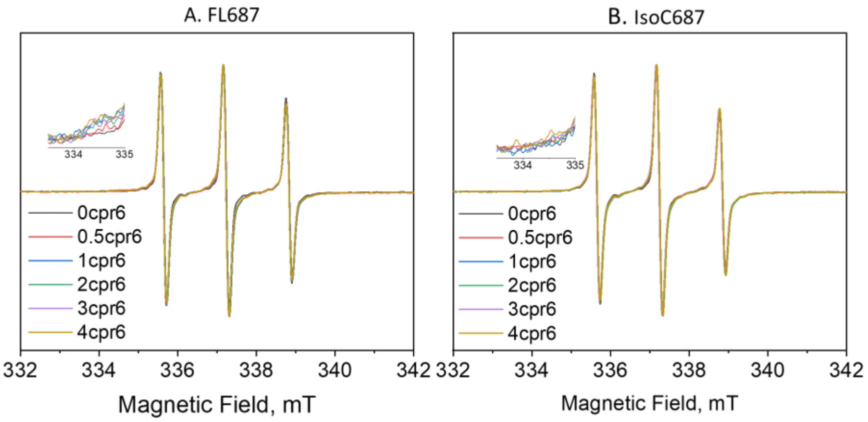

Figure S8. CW-EPR spectra as a function of the amount Cpr6 added relative to Hsp90 (molar ratio) for (A) FL687-MSL and (B) IsoC687-MSL. The inserts zoom on the spectral features of the slow-motion component.

7. Additional DEER data on FL and IsoC variants labeled at the CTD

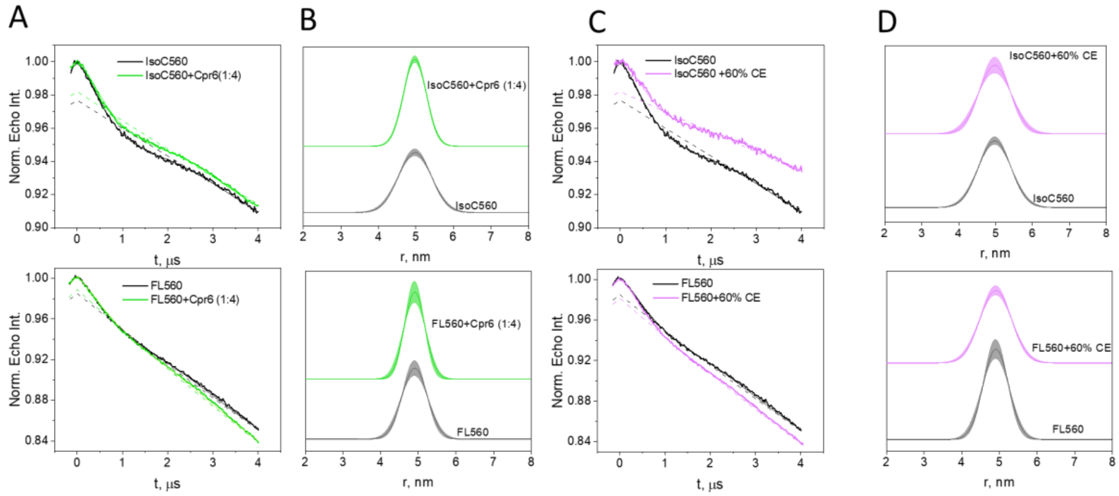

Figure S9. Primary DEER data of (A, top) IsoC560-Gd and (A, bottom) FL560-Gd with and without Cpr6 and of (C, top) IsoC560-Gd and (C, bottom) FL560-Gd with yeast cell extracts. In B and D are shown the distance distributions obtained by global fit using GLADDvu, with the background decay function in dashed lines and the fit as thin lines, shown in A and C.

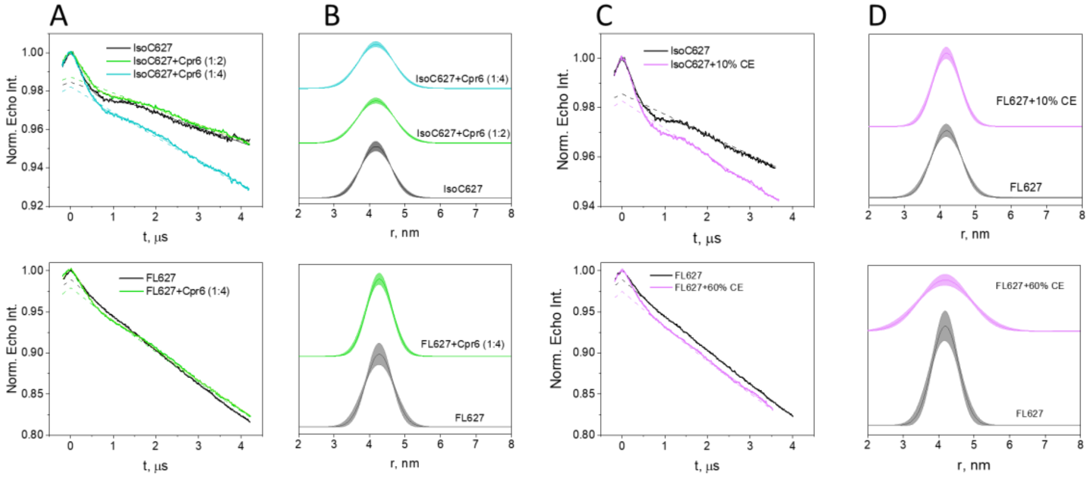

Figure S10. Primary DEER data of (A, top) IsoC627-Gd and (A, bottom) FL627-Gd with and without Cpr6 and of (C, top) IsoC627C-Gd and (C, bottom) FL627-Gd with yeast cell extracts. In B, D are shown the distance distributions obtained by global fit using GLADDvu, with the background decay function in dashed lines and the fit as thin lines shown in A and C.

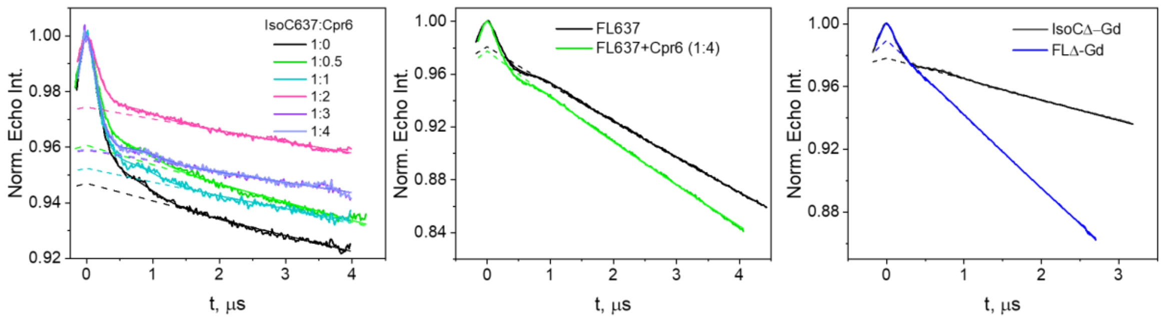

Figure S11. Primary DEER data of **Fig. 4** in the main text with the fitted data and the background correction function in solid thin and dashed lines, respectively, obtained using GLADDvu.

8. NMR measurements

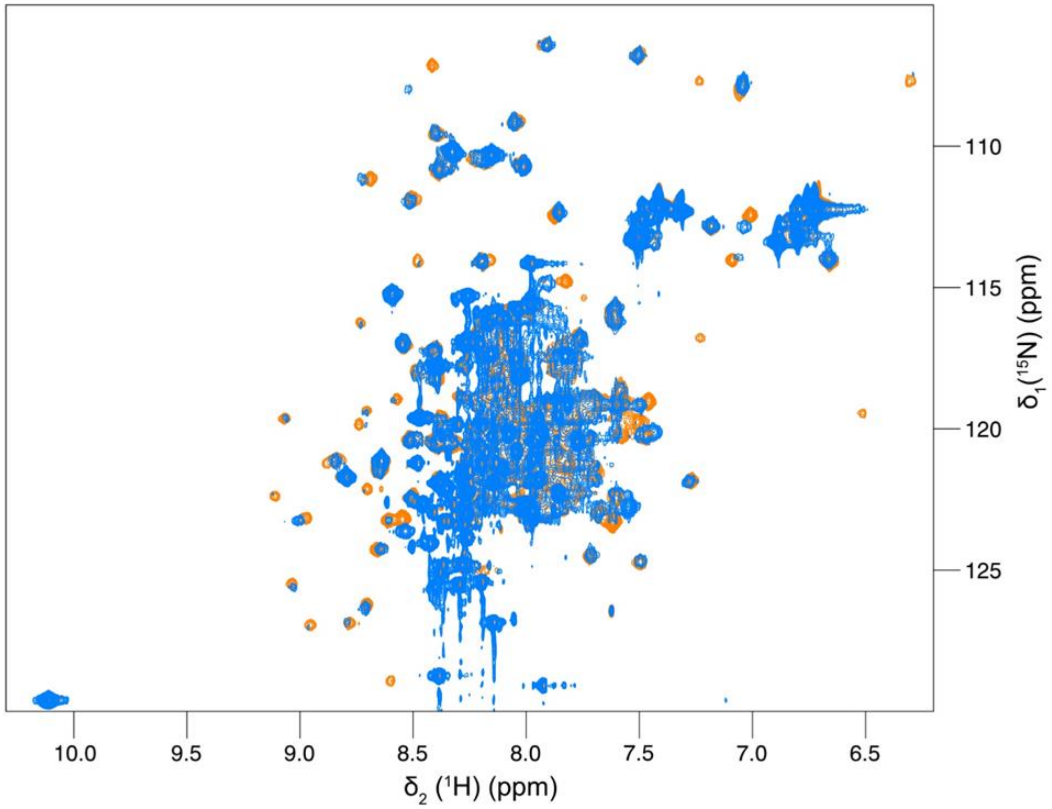

Figure S12. The $^{1}H^{15}N$ HSQC spectrum of IsoCΔ (orange) and isoC (skyblue) showing residues perturbed by tail contacts.

9. DSF and MST measurements

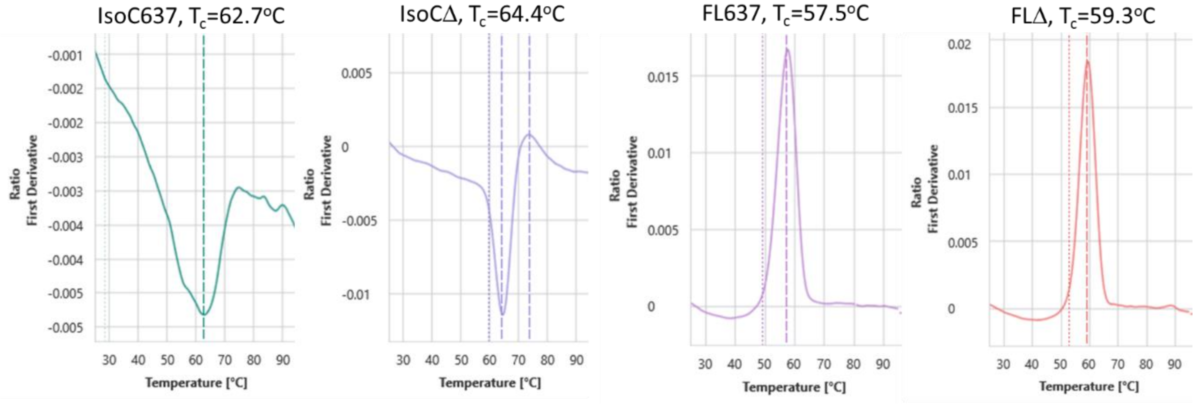

Figure S13. DSF data of IsoCΔ and FLΔ as compared to IsoC637 and FL637.

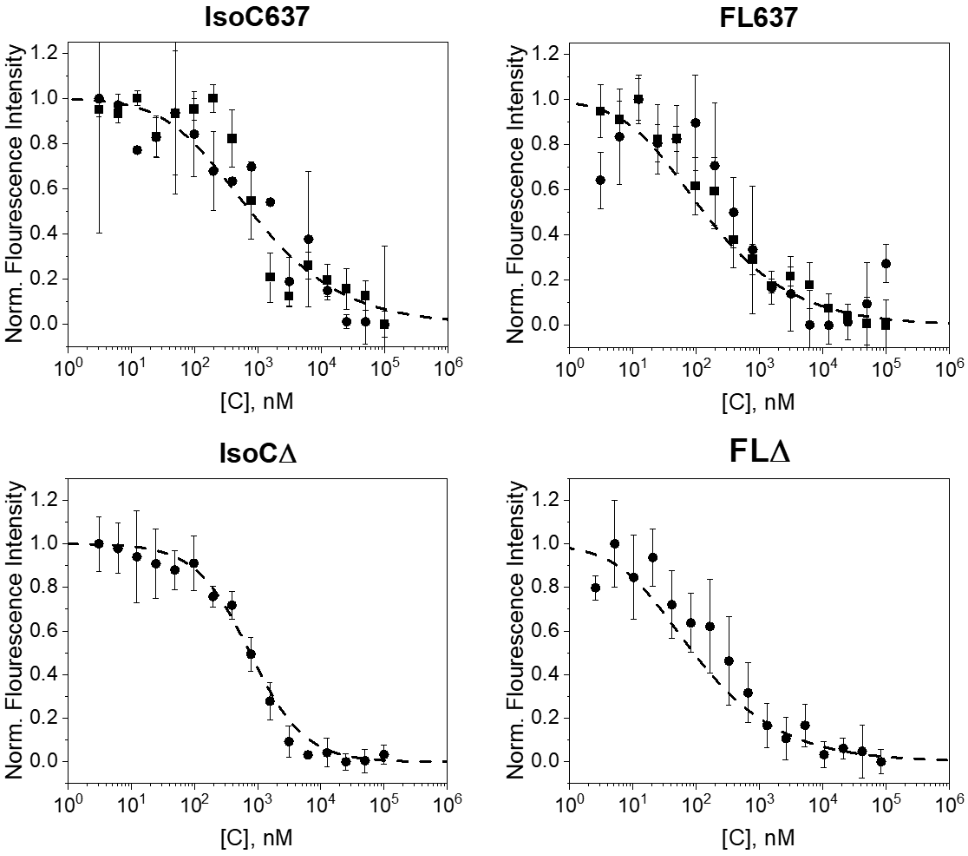

Figure S14. MST data on IsoCΔ and FLΔ as compared to IsoC637 and FL637 where [C] is the Hsp90 variant concentration. The dashed line corresponds to the fit of the data as reported earlier.[2] This model differs from the standard quadratic model by taking into account that we are dealing with a homodimer as opposed to the quadratic model that accounts for a heterocomplex. In the case of IsoCΔ this gave a rather poor fit, and the quadratic model gave a better fit. The two types of symbols refer to different experiments. **Table S4** gives the derived dimerization dissociation values $K_{\text{d-dimer}}$.

Table S4. The dimerization dissociation constant, $K_{d\text{-}dim}$, of the Hsp90 variants derived from MST measurements.

	**isoC637**	**IsoCΔ**	**FL637**	**FLΔ**
$K_{d\text{-}dim}$ / μM	0.99 ± 0.31	0.02 ± 0.01<br>0.76 ± 0.23[a]	0.16 ± 0.03	0.11 ± 0.06

[a] Obtained with quadratic model because the other model yielded a bad fit.

10. Thermodynamic model

We analyzed the data of **Fig. 4D** considering a model where one Cpr6 molecule binds to one IsoC monomer, and IsoC has two different conformations, $IsoC_a$ and $IsoC_b$, with different Cpr6 binding affinities given by $K_{d,a}$ and $K_{d,b}$, respectively.
P_a and P_b are the populations of the $IsoC_a$ and $IsoC_b$ conformations, respectively, and their ratio is given by K_0

$$K_0 = \frac{P_b}{P_a} \qquad \text{(S1)}$$

F_a is the observable, i.e. the fraction of $IsoC_a$

$$\frac{1}{F_a} = 1 + \frac{K_0[1+B_t(K_{d,a})^{-1}]}{1+B_t(K_{d,b})^{-1}} \qquad \text{(S2)}$$

where B_t is the total concentration of Cpr6, and P_t is the total monomer concentration of IsoC (100 μM). This is needed because in eq. S2 the $K_{d,a}$ and $K_{d,a}$ are given relative to P_t.
The data of **Fig. 4D** were fit to eq. (S2) to obtain two different K_d's. The fit obtained is given **Fig. S16** and is reasonable.

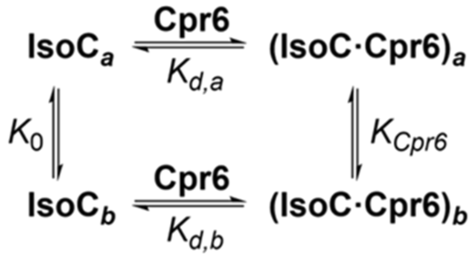

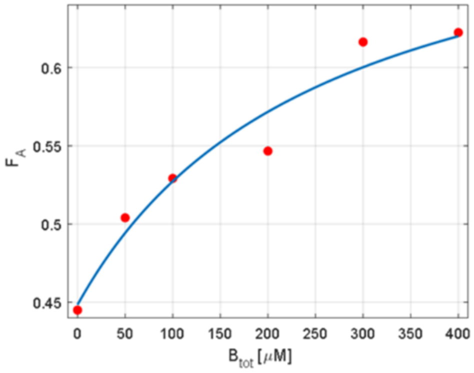

Figure S15. Thermodynamic equilibria used (left) to fit the data of **Fig. 4D**. Best fit of the data gave $K_{d,a}$= 158 ± 32 μM and $K_{d,b}$ = 528 ± 105 μM, with uncertainties estimated from variations of the $K_{d,a}$ and $K_{d,b}$ around the best fit values.

11. DEER measurements in cells and cell extracts

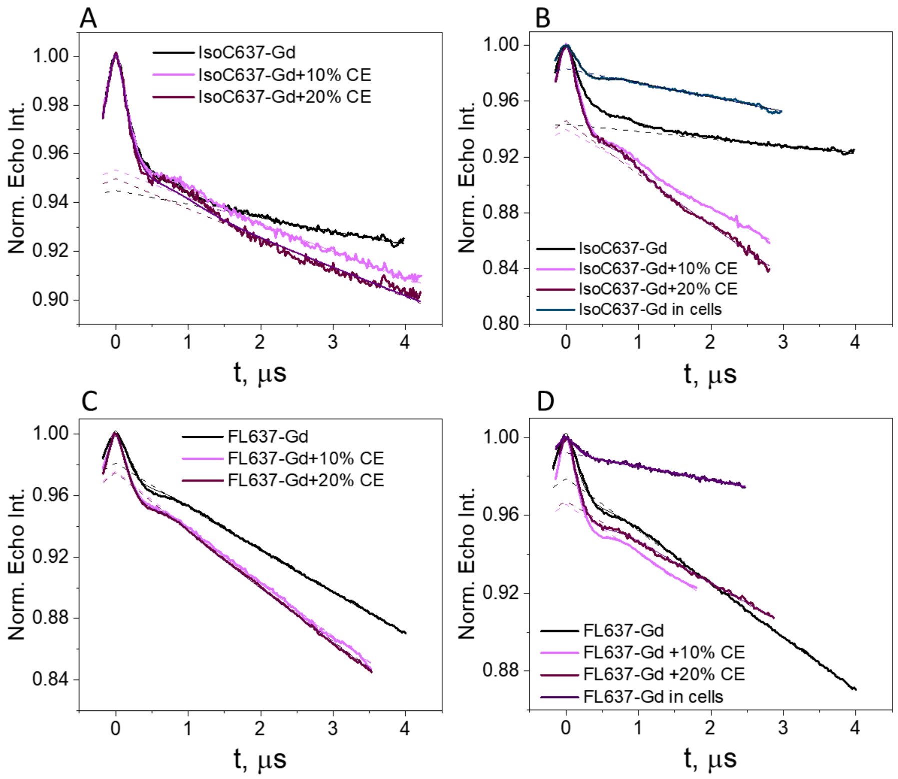

Figure S16. Primary DEER data of **Fig. 6** in the main text with the fitted data and the background correction function in solid thin and dashed lines, respectively, obtained by global fit using GLADDvu.

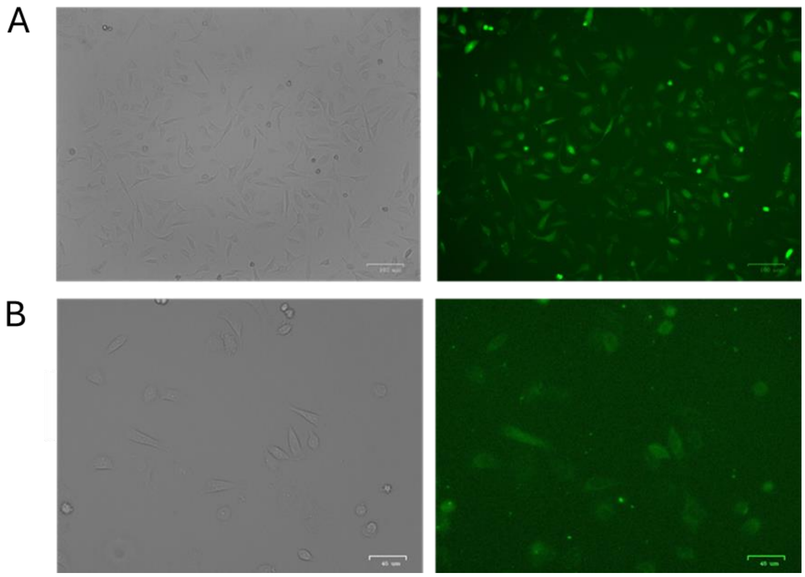

Figure S17. Optical (left) and fluorescence (right) microscopy images of (A) IsoC637 and (B) FL637 upon specific labeling with a fluorescent tag (ATTO488) after delivery into HeLa cells, scale bar 100 μm and 45 μm, respectively.

12. Pull-down experiments

Protein groups identified in pull-down experiments using the isolated Hsp90 C-terminal domain in datasets 1 and 2, annotated with fold enrichment values, TPR/MEEVD-binding classification, and functional group assignments can be found in Tables S5 and S6 (separate files in the SI)

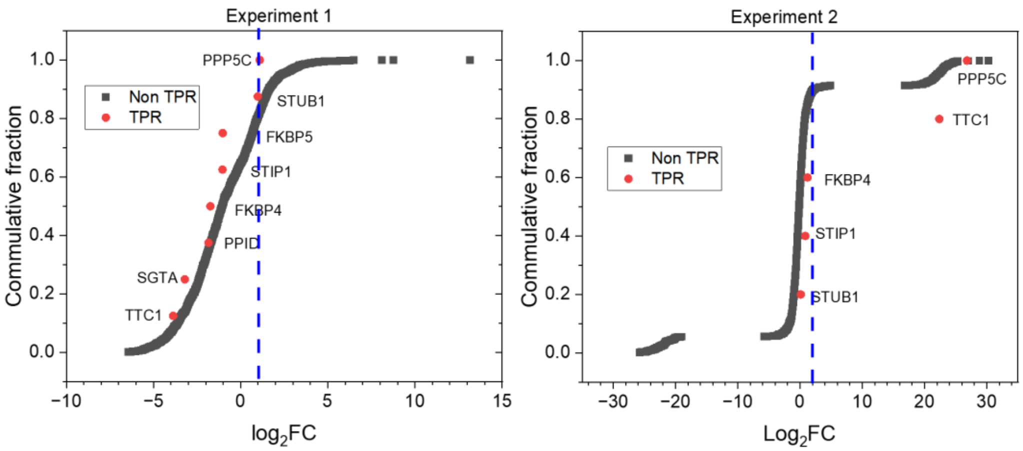

Figure S18. Cumulative distribution plots of $\log_2$FC (FC=fold change values, sample versus control) for proteins identified in pull-down experiments using the isolated Hsp90 C-terminal domain. The cumulative distribution fraction (CDF) for each individual protein is given by $CDF_i = i/N$, where i is the rank of the protein in the sorted list of $\log_2$FC, with 1 corresponding to the smallest and N the total number of proteins in the group. Two datasets are shown: a replicate-based analysis (dataset 1, left panel) and a single sample–control comparison (dataset 2, right panel). TPR-containing proteins known to bind the conserved MEEVD motif of Hsp90 are shown alongside non-TPR proteins. The vertical dashed line indicates a two-fold enrichment threshold $\log_2$FC = $\log_2$(intensity in sample/intensity in control). The plots illustrate context-dependent enrichment behavior, with TPR proteins reaching higher apparent enrichment in the single-comparison dataset and clustering at moderate enrichment values under replicate-stringent conditions (the data for these plots can be found in Tables S7 and S8 (separate files in the SI).

Non-parametric statistical comparison of the enrichment distributions in dataset 1 using Kolmogorov–Smirnov and Mann–Whitney tests applied to $\log_2$FC values (replicate averaged) did not reveal a significant global difference between TPR and non-TPR proteins (KS test, $p = 0.64$; Mann–Whitney test, $p = 0.60$), indicating that TPR proteins do not dominate the overall enrichment distribution under replicate-stringent conditions.

References

(1) Oranges, M.; Giannoulis, A.; Vanyushkina, A.; Sirkis, Y. F.; Dalaloyan, A.; Unger, T.; Su, X.-C.; Sharon, M.; Goldfarb, D. C-terminal domain dimerization in yeast Hsp90 is moderately modulated by the other domains. *Biophys. J.* **2024**, *123* (2), 172–183. DOI: 10.1016/j.bpj.2023.12.005 (accessed 2025/07/13).

(2) Mayr, C.; Richter, K.; Lilie, H.; Buchner, J. Cpr6 and Cpr7, two closely related Hsp90-associated immunophilins from Saccharomyces cerevisiae, differ in their functional properties. *J. Biol. Chem.* **2000**, *275* (44), 34140–34146. DOI: 10.1074/jbc.M005251200 From NLM.

(3) Hagelueken, G.; Ward, R.; Naismith, J. H.; Schiemann, O. MtsslWizard: In Silico Spin-Labeling and Generation of Distance Distributions in PyMOL. *Appl. Mag. Reson.* **2012**, *42* (3), 377–391. DOI: 10.1007/s00723-012-0314-0.
